# Tremor: Clinical Frameworks, Network Dysfunction and Therapeutics

**DOI:** 10.3390/brainsci15080799

**Published:** 2025-07-27

**Authors:** Emmanuel Ortega-Robles, Oscar Arias-Carrión

**Affiliations:** 1 División de Neurociencias, Clínica, Instituto Nacional de Rehabilitación Luis Guillermo Ibarra Ibarra, Mexico City 14389, Mexico; 2 Tecnologico de Monterrey, Escuela de Medicina y Ciencias de la Salud, Mexico City 14380, Mexico

**Keywords:** tremor classification, essential tremor, network oscillations, neuromodulation, AI in movement disorders

## Abstract

**Background:** Tremor is a common but diagnostically challenging movement disorder due to its clinical heterogeneity and overlapping aetiologies. The 2018 consensus introduced a two-axis classification system that redefined tremor syndromes by distinguishing between clinical phenomenology and underlying causes, and introduced new diagnostic categories, such as essential tremor plus. **Methods:** This review synthesises recent advances in the epidemiology, classification, pathophysiology, and treatment of tremor syndromes, aiming to provide an integrated and clinically relevant framework that aligns with emerging diagnostic and therapeutic paradigms. **Results:** We discuss how electrophysiology, neuroimaging, wearable sensors, and artificial intelligence are reshaping diagnostic precision. Syndromes such as essential tremor, parkinsonian tremor, dystonic tremor, task-specific tremor, orthostatic tremor, and functional tremor are examined through syndromic, aetiological, and mechanistic lenses. The limitations of current rating scales and the promise of emerging biomarkers are critically assessed. **Conclusions:** As therapeutic approaches evolve toward neuromodulation and precision medicine, the need for pathophysiologically grounded diagnostic criteria becomes more urgent. Integrating network-based frameworks, digital diagnostics, and individualised treatment holds promise for advancing tremor care.

## 1. Introduction

Tremor, a prevalent and often debilitating movement disorder, affects millions worldwide [1,2]. Traditionally viewed as a benign symptom of ageing, tremor is now recognised as a complex, multifaceted condition that spans all age groups, implicating diverse neurological disorders such as Parkinson’s disease (PD), essential tremor (ET), and dystonia. Despite decades of research, the clinical community continues to face ongoing challenges in classifying and managing this disorder due to its heterogeneous nature, varied aetiologies, and complex clinical presentations [3].

For decades, tremor classification has been based primarily on clinical observation and presumed anatomical correlates; however, these frameworks have often fallen short in capturing the full heterogeneity and pathophysiological complexity of tremor syndromes [4]. Although the 1998 consensus criteria marked a significant milestone, they did not address the nuanced differences between various tremor syndromes [5,6,7]. Essential tremor, once considered a straightforward idiopathic condition, has since evolved into a multifaceted syndrome influenced by genetic predispositions and environmental factors, underscoring the limitations of older classification systems. Advances in neuroimaging and genetic research have exposed gaps in our understanding, revealing that tremor is far more intricate than previously thought [8].

The 2018 consensus criteria introduced important updates, reflecting the growing complexity of tremor pathophysiology and offering a more flexible diagnostic framework. It includes a two-axis classification system that separates clinical phenomenology from aetiology, a stricter definition of ET, and the introduction of the ET plus category to account for cases with additional neurological signs, thereby addressing some of the limitations of previous classifications [5].

Despite the growing availability of advanced diagnostic tools, current protocols primarily rely on observational frameworks—assessing tremor by its location, frequency, and amplitude [9]. While clinical examination remains essential, it often lacks the specificity needed to distinguish between the diverse spectrum of tremor syndromes. These challenges are further compounded by the limitations of existing classification systems, which may impose a one-size-fits-all model on a clinical landscape that increasingly demands precision and personalised assessment [10].

Here, we critically examine how recent advances in clinical neurophysiology, neuroimaging, genetics, and emerging technologies are enhancing the diagnostic precision and pathophysiological understanding of tremor syndromes. This review aims to offer a contemporary and integrative framework for understanding tremor syndromes by synthesising syndromic, aetiological, and mechanistic dimensions. By critically evaluating advances in classification, network pathophysiology, diagnostic strategies, and treatment modalities, we aim to support clinicians and researchers in enhancing diagnostic accuracy, informing management, and informing future research in the field of tremor.

## 2. Methods

This review synthesises current knowledge on the epidemiology, pathophysiology, classification, diagnosis, and treatment of tremor syndromes. Although not conducted as a systematic review, we adopted a rigorous and comprehensive search strategy to ensure breadth and relevance. Searches were performed using PubMed and Scopus from inception to May 2025, without restrictions on language or publication date. We prioritised original research and review articles published within the past ten years to capture recent advances, while also incorporating seminal or highly cited older studies when pertinent to conceptual or mechanistic developments.

We employed an iterative search strategy that combined keywords and medical subject headings (MeSHs) related to tremor and its subtypes. Terms included the following: “tremor”, “essential tremor”, “parkinsonian tremor”, “dystonic tremor”, “Holmes tremor”, “cerebellar tremor”, “task-specific tremor”, “palatal tremor”, “orthostatic tremor”, “functional tremor”, and “enhanced physiological tremor”, as well as related terms such as “Parkinson’s disease”, “dystonia”, “head tremor”, “vocal tremor”, “writing tremor”, “geniospasm”, “rabbit syndrome”, “genetics”, “GWAS”, “electrophysiology”, “neuroimaging”, “MRI”, “deep brain stimulation”, “focused ultrasound”, “wearables”, “artificial intelligence”, and “machine learning”.

The reference lists of retrieved articles were manually screened to identify additional relevant sources. No formal inclusion or exclusion criteria were applied; instead, articles were selected based on their scientific quality, clinical relevance, and contribution to advancing understandings of tremor syndromes. Multiple authors independently screened titles and abstracts to ensure coverage across key domains, including aetiology, pathophysiology, clinical features, diagnostic tools, and therapeutic interventions.

The selected literature was critically appraised, and the findings were integrated into thematic sections that reflect the current consensus, controversies, and translational frontiers. Where applicable, we emphasised novel insights from neurophysiology, neuroimaging, and genetics, as well as emerging therapeutic strategies such as adaptive neuromodulation and artificial intelligence-assisted diagnosis. This narrative approach aimed to provide a structured and up-to-date synthesis of tremor research, supporting clinicians and investigators in navigating this heterogeneous and evolving field.

## 3. Epidemiology

Global prevalence studies encompassing all forms of tremor remain limited in the literature. For instance, a study by Rautakorpi et al., conducted in two rural municipalities in Finland, assessed 3080 individuals aged 40 years or older. Tremor was reported as occurring “often” or “fairly often” in 8.2% of the population, with 55% of these individuals receiving a diagnosis of ET [11]. Another study in a Brazilian town surveyed 1186 residents aged over 64 years and found a tremor prevalence of 17.4%, including 7.4% for ET, 5.6% for parkinsonian tremor, 2.8% for enhanced physiological tremor, and 1.6% attributed to other causes [12]. In a semi-urban Italian community, 706 individuals aged 50–89 years underwent neurological assessment. Movement disorders were observed in 28.0% of the sample, with tremor identified in 14.5% of cases (53.3% of all movement disorders), comprising 9.5% enhanced physiological tremor, 3.1% ET, 2.8% parkinsonian tremor, and <0.2% cerebellar tremor [13]. Similarly, in a study of 397 residents in a New York City nursing home (mean age 85.9 ± 8.5 years, range 56–104), 21% had a diagnosed movement disorder; ET accounted for 8.8% and drug-induced tremor for 3.0% of cases [14].

Although data on tremor overall remain limited, the epidemiology of essential tremor has been comparatively well characterised in the literature. ET is considered not only the most common form of tremor but also the most prevalent adult-onset movement disorder and one of the most common neurological disorders in adults [15,16]. Reported prevalence estimates for ET in general populations range from 0.32% to 1.33%, with an increase to 5.8% in individuals aged 65 years and older [1,2].

The Global Burden of Disease study by the Institute for Health Metrics and Evaluation includes the most prevalent neurological disorders but does not report specific data on tremor [17]. This gap underscores the need for robust epidemiological studies to define the global prevalence and clinical subtypes of tremor. Accurate population-based estimates could improve awareness, facilitate early recognition, and support timely diagnosis and treatment.

## 4. Consensus Criteria for Tremor Classification (2018)

The 1998 consensus criteria for tremor classification represented a significant advancement in understanding tremor syndromes, but several limitations became evident over time [5,6,7]. These limitations were particularly noticeable in the inconsistency of defining tremors based on semiology, anatomical origin, and aetiology. The definition of essential tremor as an idiopathic disorder did not adequately capture its phenotypic variability or the newly identified underlying causes. In response to these challenges, the 2018 consensus criteria introduced essential updates, reflecting the growing complexity of tremor pathophysiology and offering a more flexible diagnostic framework [5].

One of the key innovations of the 2018 consensus criteria is the introduction of a two-axis classification system. Axis 1 focuses on the syndromic diagnosis and is based on a comprehensive clinical evaluation that integrates several domains: medical history (including age of onset, family history, temporal evolution, and exposure to medications or toxins); tremor characteristics (such as body distribution, activation condition—rest, postural, or kinetic—and frequency); associated signs (including systemic features, neurologic findings, and “soft signs” such as mild dystonia or impaired tandem gait); and laboratory investigations (such as electrophysiological studies, structural and receptor imaging, and relevant biomarkers). Axis 2 addresses the underlying aetiology, which may be acquired, genetic, or idiopathic. This two-dimensional framework enables a more nuanced and dynamic approach to diagnosis, recognising that multiple aetiologies may contribute to a single tremor syndrome and that syndromic presentations can evolve without necessarily changing the underlying cause.

The redefinition of ET is a significant advancement in the new criteria. Previously regarded as an idiopathic or familial condition, it is now recognised as a heterogeneous syndrome with variable clinical features and potentially diverse underlying mechanisms. ET is now subdivided into two categories: essential tremor, referring to isolated bilateral upper limb action tremor of at least three years’ duration without additional neurological signs; and essential tremor plus, which describes cases with similar tremor features accompanied by subtle neurological signs such as impaired tandem gait, questionable dystonia, or cognitive changes. Tremors of less than three years’ duration that do not meet criteria for other syndromes are classified as indeterminate tremor. This updated framework acknowledges the phenotypic variability of ET and supports further exploration of diverse underlying mechanisms, including genetic contributions [6].

The concept of syndromic evolution is another important feature. Tremor syndromes are recognised as capable of progressing from one classification to another without altering their underlying aetiology. For example, an isolated resting tremor may evolve into a parkinsonian tremor, indicating that the initial tremor was likely of parkinsonian origin. Similarly, essential tremors may progress to parkinsonian tremors, suggesting the coexistence of or progression to a new diagnosis. This approach emphasises the importance of longitudinal assessment, allowing clinicians to monitor the clinical course of tremor and adjust diagnoses and treatments as needed.

In addition to these changes, the 2018 consensus criteria expanded the tremor classification framework to encompass other rhythmic movement disorders, thereby improving diagnostic clarity. For example, myorhythmia—a slow, rhythmic movement typically involving cranial or limb muscles—is now recognised as part of the spectrum of tremor syndromes. This expanded classification enhances clinical recognition and supports more accurate diagnosis and management of tremor-related conditions [3].

Finally, the introduction of the indeterminate tremor category provides a proper provisional diagnosis for cases that do not yet meet the full criteria for a defined tremor syndrome. This designation enables clinical re-evaluation over time, supporting a more flexible, individualised, and dynamic approach to tremor diagnosis and classification.

While the 2018 consensus criteria represented a substantial step forward in standardising tremor classification, several limitations have emerged. Notably, the introduction of the “essential tremor plus” (ET plus) category—defined by the presence of soft neurological signs—has sparked controversy due to its lack of operational clarity and inter-rater reliability. The criteria remain insufficiently validated across diverse populations and clinical settings, limiting their generalisability. Moreover, the current framework does not adequately incorporate mechanistic biomarkers or recent advances in network neuroscience, electrophysiology, and genetics, which increasingly reveal the heterogeneity of tremor syndromes. These gaps suggest the need for continued refinement and expansion of the classification system.

## 5. Classification of Tremors

**Figure 1:**
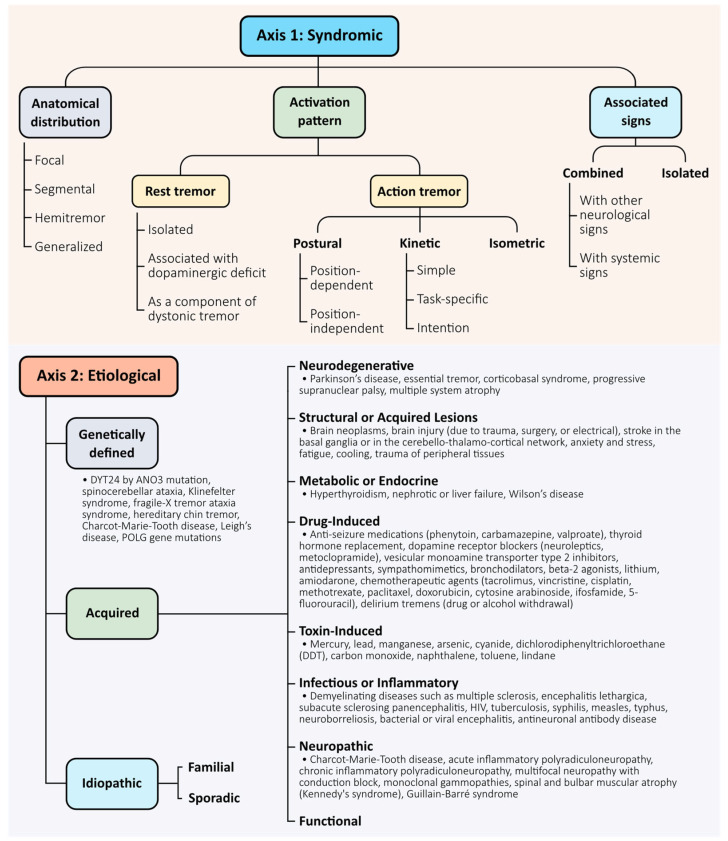
Two-axis classification framework for tremor syndromes. Tremors are classified along two complementary axes, as proposed by the 2018 consensus criteria [5]. Axis 1 defines the clinical syndrome and includes three core descriptors: anatomical distribution (focal, segmental, hemitremor, generalised), activation pattern (rest, postural, kinetic, intention, task-specific, or isometric), and associated neurological signs (isolated vs combined). Axis 2 specifies the underlying aetiology, which may be acquired (e.g., neurodegenerative, toxic, metabolic), genetically defined (monogenic or syndromic), or idiopathic—including both familial and sporadic forms in the absence of a confirmed cause. This dual-axis model supports structured clinical reasoning and accommodates diagnostic evolution over time.

The 2018 consensus introduced a two-axis framework to classify tremors based on clinical presentation (Axis 1) and underlying aetiology (Axis 2) [5]. This system provides a structured foundation for syndromic diagnosis and remains central to clinical decision-making and treatment planning. Axis 1 characterises tremor by its anatomical distribution (focal, segmental, hemitremor, or generalised), activation pattern (rest, postural, kinetic, or intention), and associated signs (isolated vs. combined tremor). Axis 2 considers whether the tremor is acquired, genetic, or idiopathic. Together, these axes help clinicians navigate the diagnostic complexity of tremor syndromes (Figure 1).

While the classification system is useful, we acknowledge its limitations and emphasise its evolving nature. The categorisation does not fully capture the mechanistic diversity of tremor, and several entities—particularly essential tremor plus—remain controversial. Additionally, classification may shift over time as clinical features evolve or as mechanistic understanding deepens.

Importantly, emerging technologies such as high-resolution imaging, electrophysiological mapping, and artificial intelligence are beginning to reshape the classification of tremors based on their underlying network dynamics, rather than solely on phenomenological traits. In this context, the current classification should be seen as a clinical scaffold to be enhanced—not replaced—by mechanistic biomarkers and data-driven approaches.

Looking forward, a key area for improvement involves integrating a third dimension—a “mechanistic axis”—to complement the current syndromic and aetiological framework [18]. This axis could reflect circuit-level dysfunctions, as informed by neuroimaging, electrophysiology, and digital biomarkers, and may enable more precise subtype classification and targeted therapeutic interventions. Furthermore, incorporating artificial intelligence-based phenotyping and large-scale genetic data could support the development of predictive and personalised diagnostic models. A dynamic and multidimensional classification system would better reflect the biological diversity of tremor and support its evolving clinical management.

With this perspective, the subsequent section focuses on current models of tremor pathophysiology, which offer critical insight into the circuits and mechanisms that transcend clinical subtypes and point toward future classification strategies grounded in systems neuroscience.

## 6. Pathophysiology of Tremor

**Figure 2:**
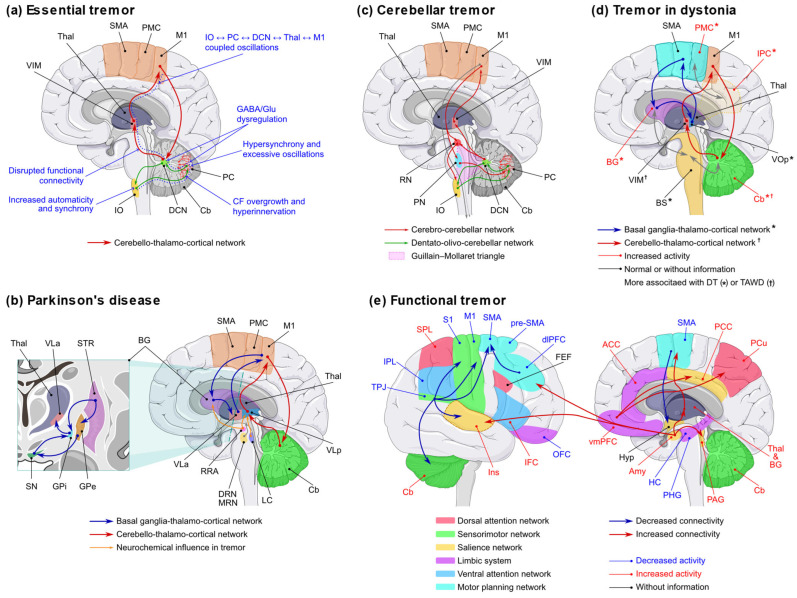
Neural circuits and pathophysiological mechanisms underlying tremor syndromes. (**a**) Four main hypotheses have been proposed for ET: the cerebellar oscillator hypothesis (intrinsic cerebellar rhythmicity), the central oscillatory network hypothesis (synchronised activity across a motor network), the GABAergic/glutamatergic dysfunction, and the cerebellar decoupling hypothesis (disrupted connectivity affecting motor control). All involve the cerebello-thalamo-cortical circuit. These are not mutually exclusive and may collectively contribute to tremor. (**b**) In PD, the “dimmer-switch” model suggests that the basal ganglia trigger tremor onset, while the cerebello-thalamo-cortical circuit sustains it. Striatal inhibition of the GPi activates the VLa, relaying signals to the cortex, where both circuits converge. Dopaminergic (RRA), noradrenergic (LC), and serotonergic (RN) systems also contribute. (**c**) Cerebellar tremor arises from disruption of the cerebellar circuits involved in motor coordination, particularly the cerebro-cerebellar and dentato-olivo-cerebellar pathways. Lesions affecting the Guillain–Mollaret triangle can lead to aberrant oscillatory activity, contributing to the generation of tremor. (**d**) DT is a network disorder involving both cerebello– and basal ganglia–thalamo–cortical pathways, with the latter predominating. TAWD resembles essential tremor and is mainly driven by the cerebello–thalamo–cortical circuit, with minimal basal ganglia involvement. (**e**) In functional tremor, evidence suggests dysfunction across networks involved in salience, interoception, agency, emotion regulation, and attention, characterised by altered activity and connectivity in regions such as the limbic system, motor cortex, and self-referential areas. *Abbreviations:* ACC, anterior cingulate cortex; Amy, amygdala; BG, basal ganglia; BS, brainstem; Cb, cerebellum; DCN, deep cerebellar nuclei; dlPFC, dorsolateral prefrontal cortex; DRN and MRN, dorsal and median raphe; DT, dystonic tremor; ET, essential tremor; FEF, frontal eye fields; GPi and GPe, internal and external globus pallidus; HC, hippocampus; Hyp, hypothalamus; IFC, inferior frontal cortex (gyrus); Ins, insula; IO, inferior olive; IPC, inferior parietal cortex; IPL, inferior parietal lobule; LC, locus coeruleus; M1, primary motor cortex; OFC, orbitofrontal cortex; PAG, periaqueductal grey; PCC, posterior cingulate cortex; PC, Purkinje cells; PCu, precuneus; PD, Parkinson’s disease; PHG, parahippocampal gyrus; PMC, premotor cortex; PN, pontine nucleus; RRA, retrorubral area; RN, red nucleus; S1, primary somatosensory cortex; SMA, supplementary motor area; SN, substantia nigra; SPL, superior parietal lobule; TAWD, tremor associated with dystonia; Thal, thalamus; TPJ, temporoparietal junction; VIM, ventrointermediate nucleus of thalamus; VLa and VLp, anterior and posterior ventrolateral nucleus of the thalamus; vmPFC, ventromedial prefrontal cortex; VOp, ventralis oralis posterior nucleus of thalamus.

Tremor is increasingly recognised as a network disorder arising from dysregulated oscillatory activity within central motor circuits. Although tremor syndromes, such as parkinsonian tremor, essential tremor, tremor in dystonia, and Holmes tremor, are clinically and aetiologically heterogeneous, converging evidence suggests that they commonly involve the cerebello-thalamo-cortical circuit, albeit through distinct mechanisms and circuit dysfunctions (Figure 2) [18,19,20].

In Parkinson’s disease, tremor is typically characterised by a resting phenotype and exhibits a variable response to dopaminergic therapy [21]. Recent models propose that tremor generation and maintenance involve a dynamic interaction between basal ganglia and cerebellar circuits. Specifically, the “dimmer-switch” model posits that the basal ganglia initiate tremor episodes, while the cerebellum circuit sustains them [22]. This is supported by electrophysiological findings showing transient increases in alpha/low-beta activity in the subthalamic nucleus (STN) at tremor onset and sustained tremor-frequency oscillations in the STN and motor cortex during tremor maintenance [23]. Neuroimaging studies also highlight that dopamine-resistant tremor is associated with increased cerebellar activity, whereas dopamine-responsive tremor involves altered thalamic activity, suggesting the existence of distinct subcortical mechanisms [24].

In essential tremor, long considered a benign, monosymptomatic disorder, pathophysiological models now suggest that cerebellar dysfunction is central to its genesis. Two non-mutually exclusive hypotheses have emerged. The cerebellar oscillator hypothesis suggests that tremor is driven by excessive cerebellar activity—particularly within sensorimotor lobes—while the cerebellar decoupling hypothesis proposes that tremor results from structural and functional disconnection between the cerebellum and its projection targets, such as the dentate nucleus and thalamus [25,26]. Supporting evidence includes reduced cerebellar white matter integrity, Purkinje cell pathology, and tremor attenuation following deep brain stimulation (DBS) [27,28,29].

Tremor in dystonia appears to arise from more heterogeneous mechanisms. Functional imaging reveals overlapping cerebellar activation patterns associated with essential tremor, while also highlighting widespread network abnormalities that involve cortical, basal ganglia, and cerebellar circuits [30]. Reduced self-inhibition in the basal ganglia and premotor areas may contribute to the generation of tremors [31]. Clinical characteristics, such as the quality of tremor (e.g., jerky vs. sinusoidal), may help to distinguish dominant circuit involvement in individual patients [32].

In Holmes tremor, lesion network mapping has revealed that causative lesions—despite their anatomical variability—consistently affect a shared functional network comprising the red nucleus, globus pallidus internus (GPi), thalamus, cerebellum, and pontomedullary junction. This supports the idea that tremor can emerge from disruption at multiple nodes within a shared neural architecture [33].

A recent multimodal study by Goede et al. identified a convergent tremor-suppressing network involving the motor cortex, cerebellum, and dentato-rubro-thalamic tract, reinforcing the concept of tremor as a network disorder. This supports a shared anatomical substrate underlying diverse tremor syndromes [34].

Recent findings have drawn attention to the zona incerta (ZI) as a critical structure in the pathophysiology of tremor, particularly in PD. The ZI, located in the subthalamic area, functions as an integrative relay between the basal ganglia, thalamus, and motor cortex. Electrophysiological and imaging studies in both human and animal models suggest that the ZI is not merely a passive anatomical bridge but an active participant in oscillatory network dynamics [35,36,37].

ZI neurons exhibit widespread functional connectivity with the motor cortex, prefrontal cortex, thalamic nuclei, midbrain, and multiple basal ganglia structures, indicating their capacity to synchronise and modulate activity across the tremor network. The concept of re-entrant connectivity—whereby cortical and subcortical circuits mutually influence each other via the ZI—has gained traction as a mechanism underlying tremor generation and propagation [37].

The role of the ZI may be particularly relevant for dopamine-resistant tremor phenotypes and tremors with complex or overlapping features. The emerging evidence suggests that dysfunction within the basal ganglia–ZI–thalamocortical circuit may be central to tremor pathogenesis in PD and potentially in other syndromes.

While central mechanisms dominate in pathological tremors, peripheral components, including the mechanical properties of limbs and reflex pathways, can modulate tremor amplitude, especially in enhanced physiological tremor. Feedback delays and spinal excitability may amplify oscillations when neuromuscular control loops are destabilised [20].

Figure 2 illustrates the pathophysiological mechanisms underlying tremor in ET, PD, cerebellar tremor, tremor in dystonia, and functional tremor. Overall, the pathophysiology of tremor reflects a complex, syndrome-specific interplay between aberrant central oscillators and network dysfunction. These insights have led to the proposal of a third axis of tremor classification, based on pathophysiological mechanisms, to complement existing clinical and aetiological frameworks. However, its adoption will depend on further validation, standardisation, and integration with clinical workflows [18].

## 7. Diagnostic Approach

**Figure 3:**
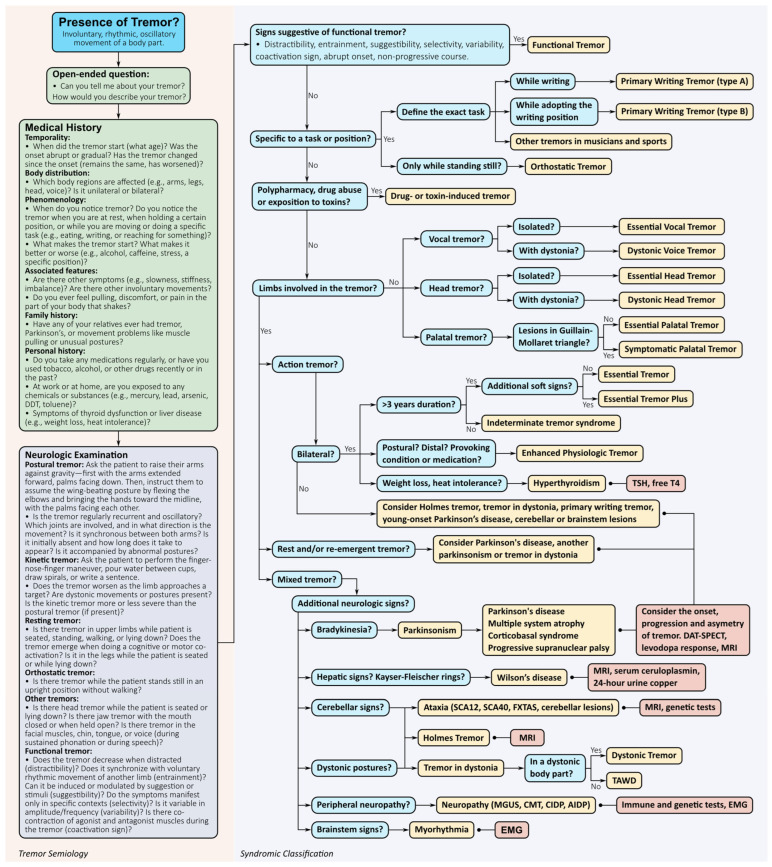
Clinical algorithm for the syndromic classification and differential diagnosis of tremor. This diagnostic flowchart outlines a structured clinical approach for patients presenting with tremor. Initial assessment includes confirmation of tremor phenomenology—defined as involuntary, rhythmic, oscillatory movement—and a detailed history of symptom onset, distribution, and exposure to medications or toxins. Neurological examination distinguishes tremor types (rest, postural, kinetic, intention) and identifies associated features such as dystonia, cerebellar signs, parkinsonism, or sensory neuropathy. Clues such as hepatic signs, including Kayser–Fleischer rings, are essential to prompt evaluation for Wilson’s disease, particularly in younger patients with mixed or atypical features. Subsequent diagnostic steps incorporate phenotypic refinement and targeted testing—such as serological, imaging, electrophysiological, or genetic—supporting classification into essential tremor, dystonic tremor, parkinsonian tremor, functional tremor, or tremors of metabolic, structural, or neurodegenerative origin.

The diagnosis of tremor requires a structured, multistep clinical process aimed at identifying its phenomenological characteristics and, if possible, the underlying cause. Given the heterogeneity of aetiologies and presentations, an accurate diagnosis relies on a comprehensive medical history, targeted neurological examination, judicious use of ancillary tests, and, in uncertain cases, longitudinal follow-up (Figure 3) [9].

### 7.1. Medical History and Physical Examination

The initial assessment begins with a detailed clinical history. Open-ended questions enable patients to describe their symptoms in their own terms, while follow-up questions clarify the onset, progression, duration, and distribution of the tremor. The clinician should determine whether the tremor is present at rest, during sustained posture, or with movement, and whether it improves with specific sensory tricks or substances such as alcohol. Associated features, including gait instability, falls, stiffness, changes in speech, or pain, may suggest additional neurological involvement. A family history of tremor, Parkinson’s disease, dystonia, or ataxia is important to assess potential hereditary syndromes. Inquiry into medication use, exposure to toxins, and symptoms of systemic illness, such as thyroid dysfunction, hepatic disease, or peripheral neuropathy, is crucial for identifying reversible causes [9,38].

### 7.2. Neurologic Examination

Neurological examination is essential for characterising the type of tremor, identifying additional neurological signs, and guiding diagnosis and further testing. Postural tremor is assessed with the arms extended, while kinetic tremor is observed during goal-directed activities such as finger-to-nose testing, writing, and spiral drawing. Tremor at rest is typically evaluated while the patient is seated with fully supported limbs. The examiner should note tremor symmetry, amplitude, frequency, direction, latency (reemergence after posture), and whether it affects additional areas such as the head, voice, jaw, or legs. Special attention should be given to signs of parkinsonism (bradykinesia, rigidity, masked facies), cerebellar dysfunction (dysmetria, ataxia), dystonia (abnormal postures, null points), or sensory neuropathy (distal weakness, areflexia) [9].

Simple clinical strategies can improve diagnostic specificity. For example, the presence of a *geste antagoniste*, in which a specific manoeuvre, such as light touch, temporarily suppresses the tremor, is highly suggestive of dystonia [39,40].

Clinicians should also evaluate for entrainment, distractibility, suggestibility, marked variability, sudden onset, or spontaneous remissions, as these signs are indicative of functional tremor [41]. It is important not to prematurely label functional signs without longitudinal confirmation, as functional overlay can obscure underlying neurodegenerative disease [38].

### 7.3. Phenotype-Based Syndromic Classification

Once the tremor has been clinically characterised, the next step involves assigning a syndromic classification. Figure 3 presents a diagnostic approach based on the most frequent and clinically relevant signs to guide the identification of specific tremor syndromes. For example, essential tremor is characterised by a bilateral postural or kinetic tremor of the upper limbs that lasts at least three years, in the absence of other neurological signs. Alcohol responsiveness and handwriting impairment are common supporting features [42]. ET may later involve the head or voice, but rarely begins in those regions. If additional soft signs are present (e.g., impaired tandem gait, questionable dystonic posturing), the diagnosis of ET plus may be considered; however, it is recommended that this label be applied cautiously and that patients be re-evaluated longitudinally before final classification [39].

Other phenotypes include enhanced physiological tremor (high-frequency, low-amplitude, often medication- or anxiety-induced), parkinsonian tremor (rest tremor with a frequency of 4–6 Hz, typically asymmetric, and often re-emerging during posture after a latency), tremor in dystonia (irregular amplitude and variable frequency, associated with dystonic postures or muscle contractions), cerebellar tremor (intention-predominant, associated with dysmetria or ataxia), palatal tremor (1–3 Hz, rhythmic movements of the soft palate), and Holmes tremor (irregular tremor combining rest, postural, and intention components, usually post-lesional). Task-specific tremors, such as primary writing tremor or musician’s tremor, are forms of focal dystonia that emerge exclusively during specific skilled tasks [3,9].

### 7.4. Ancillary Testing

Laboratory studies, neuroimaging and genetic testing are guided by clinical suspicion rather than being used routinely [43]. Basic laboratory testing is advised for most patients, especially those with bilateral isolated tremor syndromes, to identify potentially reversible causes such as thyroid dysfunction, hepatic or renal failure, abnormal glucose metabolism, or electrolyte disturbances. Specific clinical signs and symptoms may guide further laboratory evaluations. For instance, in patients presenting with mixed tremor along with dystonia, parkinsonism, ataxia, psychiatric, or cognitive symptoms—particularly in younger individuals or those with a positive family history—measurement of serum ceruloplasmin levels and 24 h urinary copper excretion is recommended to evaluate for Wilson’s disease [9].

Neuroimaging, particularly brain MRI, is recommended when the tremor presents with atypical features, such as sudden onset, stepwise progression, or unilateral distribution, or when there is a family history of movement disorders accompanied by cognitive or psychiatric symptoms. MRI is mainly used to detect structural lesions in the basal ganglia, cerebellum, or brainstem that could suggest an underlying neurodegenerative or acquired disorder. However, routine imaging is not recommended for patients with typical isolated tremor syndromes unless these red flags are present. Dopamine transporter imaging can support differentiation between Parkinson’s disease and essential tremor in ambiguous presentations, but should not be used as a standalone diagnostic tool [18,43].

Genetic testing should be reserved for cases with combined tremor syndromes and a relevant family history or associated cognitive, psychiatric, or neuropathic features. This is due to the lack of known pathogenic genes for isolated tremor syndromes. When indicated, genetic testing may include targeted gene panels, whole-exome or whole-genome sequencing, depending on the suspected condition. Collaboration with a clinical geneticist is advised to ensure appropriate selection and interpretation of genetic tests [43].

Surface electromyography (EMG) and accelerometery provide objective measurements of tremor frequency and amplitude. These tools help differentiate tremor from other movement disorders, such as myoclonus, asterixis, or clonus, and can aid in distinguishing between overlapping tremor syndromes, including Parkinson’s disease, essential tremor, dystonic tremor, and functional tremor [10].

### 7.5. Longitudinal Reassessment

Diagnostic uncertainty is common in early or atypical cases. Marsili et al. propose that instead of forcing premature syndromic labelling, clinicians should assign a diagnosis of “indeterminate tremor” when features are insufficient to meet established criteria. Regular follow-up over time, with repeated examination and evolving symptom review, may reveal new signs that support a definitive diagnosis. This approach reduces the risk of misclassification and unnecessary testing while allowing time for natural history to clarify the underlying disorder [38].

## 8. Rating Scales and Clinical Assessment Tools

Clinical scales are essential tools for screening and evaluating tremor, enabling clinicians and researchers to quantify severity, assess functional impact, and monitor changes over time. Before the 1990s, there were no standardised scales with validated clinimetric properties for tremor assessment. Since then, several instruments have been developed and evaluated, particularly for essential tremor and Parkinson’s disease. These scales can be broadly grouped into categories that assess tremor severity, disability, activities of daily living (ADLs), quality of life, and screening [44,45].

The Fahn–Tolosa–Marín Tremor Rating Scale is among the most widely used instruments for evaluating tremor. It assesses rest, postural, and kinetic tremor across anatomical regions, along with performance tasks such as writing and drawing, and includes assessments of ADL and global impressions by both patients and examiners. Although this scale demonstrates good inter- and intra-rater reliability and is sensitive to treatment-related changes, it exhibits a ceiling effect in patients with severe tremors, and may not reliably assess rest tremor in disorders dominated by action tremors, such as ET. It has also been noted that the scale may not fully capture the patient’s perspective [44,46,47].

In response to these limitations, the Tremor Research Group developed the Essential Tremor Rating Assessment Scale (TETRAS), which omits rest tremor from its evaluation and incorporates higher amplitude thresholds for its upper score categories. TETRAS comprises performance and ADL subscales with high internal consistency and inter-rater reliability, even among untrained raters. It is well suited for longitudinal studies and clinical trials, and correlates strongly with objective tremor measurements [48].

The Bain and Findley Clinical Tremor Rating Scale is another commonly used instrument. It assesses tremor severity in the head, voice, and limbs using a 0–10 scale. Its ease of administration is an advantage, although variability in reliability across different body regions has been reported. The accompanying Spirography Scale evaluates spiral drawings to provide a visual and quantitative representation of action tremor. While offering fine gradations, its sensitivity to clinical change remains limited at the extremes of severity and has shown a weak correlation with ADL [49,50].

The WHIGET Tremor Rating Scale was initially developed for distinguishing essential tremor from enhanced physiologic tremor. Its first version, designed for screening, includes a diagnostic algorithm based on postural and kinetic tremor features. It has demonstrated strong inter-rater reliability and sensitivity to disease progression, and shows good agreement with the TETRAS scale. However, strict diagnostic thresholds may exclude individuals with milder tremor, and limited standardisation has restricted its broader clinical use. A revised version has since improved its utility in distinguishing pathological tremor from physiological variants [45,51,52].

Beyond tremor severity, instruments targeting functional disability and quality of life provide valuable complementary information. The Bain and Findley Tremor ADL Scale focuses on upper limb function and correlates well with other clinical scales, showing moderate sensitivity to therapeutic interventions. Quality of life assessments are typically conducted with the Quality of Life in Essential Tremor (QUEST) questionnaire, the first ET-specific tool of its kind. While QUEST offers acceptable test–retest reliability and may be more sensitive to change than generic health status instruments such as the SF-36, its acceptability has been poor due to it being time-consuming [44,49].

The interpretation of rating scale scores must consider the nonlinear relationship between numerical ratings and actual tremor amplitude. The Weber–Fechner law indicates that perceived changes in tremor correspond to logarithmic changes in physical amplitude, meaning that equal score changes on a linear scale may reflect significantly different clinical realities [45].

While rating scales remain the standard for tremor evaluation due to their practicality, validation history, and relevance to patient-centred outcomes, emerging technologies offer objective quantification of tremor characteristics. Devices such as linear accelerometers, gyroscopic transducers, and graphic tablets, though primarily used in research settings, provide critical data on tremor amplitude, frequency, and patterns. These tools represent the future of tremor evaluation, enabling more precise diagnosis and personalised treatment strategies [45,53,54].

## 9. Tremor Syndromes

Tremor syndromes can be meaningfully grouped, according to their predominant clinical manifestation or presenting features (Axis 1), into action or rest tremors, focal tremors, task- or posture-specific tremors, orthostatic tremors, tremors with prominent additional signs, and other forms [5]. This phenomenology-based approach facilitates clinical recognition, differential diagnosis, and appropriate management [3,9]. In the following subsections, each tremor syndrome will be examined in greater detail, encompassing, when possible, its epidemiology, aetiology, neuropathological findings, pathophysiological mechanisms, clinical features, diagnostic approach, and current treatment strategies.

### 9.1. Essential Tremor and Essential Tremor Plus

Essential tremor is the most common neurological movement disorder, manifesting as a postural and/or kinetic tremor primarily affecting the upper limbs at frequencies between 4 and 12 Hz. It can also involve the head and voice, with progression leading to varying levels of functional impairment [15,16,55,56].

#### 9.1.1. Epidemiology

A substantial body of literature has examined the epidemiology of ET in comparison to other tremor syndromes. However, considerable heterogeneity among studies has led to wide variation in pooled prevalence estimates. Two meta-analyses have attempted to quantify the global prevalence of ET. Song et al. analysed 29 studies from 13 countries and reported a prevalence of 0.32% across all ages, increasing to 2.87% among individuals over 80 years [1]. In contrast, Louis and McCreary reviewed 42 studies spanning 23 countries and estimated a global prevalence of 1.33%, with rates rising to 5.79% in those over 65 years of age [2]. The condition appears to affect men and women similarly, although earlier studies reported slight gender differences [55]. Although it is also considered the most common tremor disorder in children aged 4 to 17 years, its global prevalence in paediatric populations remains low and poorly characterised [57]. One study reported a prevalence of 0.41% among adolescents [58]. Notably, many cases remain undiagnosed; in some population-based studies, up to 92.8% of individuals with ET were unaware of their diagnosis prior to study participation, indicating under-recognition in healthcare settings [59].

#### 9.1.2. Genetics

Although ET is often familial (with 30–70% of patients reporting a positive family history), the genetics of ET remain unclear. Individuals with a first-degree relative with ET have a 4.7-fold increased risk [57,60]. Numerous genetic loci have been proposed, including variants in genes such as *FUS*, *SORT1*, *SCN4A*, *NOS3*, *KCNS2*, *HAPLN4/BRAL2*, and *USP46*, though replication of findings is inconsistent. A 2020 meta-analysis highlighted *LINGO1* and *STK32B* as the two loci with the most substantial evidence [61]. More recent genome-wide studies have identified additional single-nucleotide variants, such as *CA3* and *CPLX1*, though validation in diverse populations is pending [62,63]. Recent hypotheses suggest that the lack of broader genetic discoveries may stem from the involvement of non-classical genetic mechanisms [64]. As such, genetic testing is not currently employed in clinical practice but remains a target for future investigation.

#### 9.1.3. Neuropathological and Pathophysiological Findings

ET is primarily linked to dysfunction within the cerebello-thalamo-cortical circuit, as demonstrated by neuroimaging and electrophysiological studies [65,66]. However, the precise mechanisms remain debated.

The neurodegenerative hypothesis suggests ET is a slowly progressive cerebellar disorder, supported by some postmortem and imaging studies, but contradicted by others. Histological findings of Purkinje cell degeneration have been both reported and refuted [55,67]. Recent evidence highlights consistent structural and functional abnormalities in the cerebellar cortex, reinforcing the view that ET is a cerebellar Purkinje cell pathology rather than a disorder driven by dysfunction of the inferior olive [68].

Related to this hypothesis, the cerebellar decoupling hypothesis highlights structural and functional disconnection between the cerebellum and its projection targets, including the dentate nucleus and thalamus, as key contributors to tremor generation. These findings are further substantiated by microstructural imaging studies, which indicate a reduced integrity of cerebellar white matter tracts in patients with essential tremor, and by experimental evidence from animal models demonstrating that disrupted synaptic pruning in Purkinje cells can induce cerebellar oscillations and tremors [18].

The central oscillatory network hypothesis posits that tremor originates from synchronised activity in a distributed neural network, specifically involving the cerebellum, inferior olive, thalamus, and motor cortex. The coherence between EEG and EMG signals, along with supporting evidence from neuroimaging studies, reinforces this model [55,69,70].

The cerebellar oscillator hypothesis posits that ET originates from aberrant oscillatory activity within the cerebellum itself. Specifically, intrinsic cerebellar circuits, particularly involving Purkinje cells and the deep cerebellar nuclei, are thought to generate tremor-related rhythmic discharges. This model emphasises the role of pathological cerebellar rhythms as the primary generator of tremor, without necessarily involving broader network dysfunction [18].

Another theory, the GABAergic hypothesis, posits that impaired GABA-A receptor signalling leads to the disinhibition of cerebellar output and the rhythmic activation of motor pathways. Supporting evidence includes the tremor phenotype of GABA-A α1 subunit knockout mice, efficacy of GABAergic agents, and the linkage of GABA-related genes to ET. Additionally, dysfunction in glutamatergic signalling, particularly via δ2 receptors, may contribute to tremor generation [55,69].

These hypotheses underscore the complexity of ET and support the notion that it is a heterogeneous disorder involving both structural and functional cerebellar abnormalities, possibly within a broader context of network dysregulation.

#### 9.1.4. Clinical Features

Essential tremor presents with a heterogeneous range of clinical features encompassing both motor and non-motor symptoms. The hallmark of ET is a bilateral kinetic tremor of the upper limbs, typically observed during activities such as eating, drinking, or writing, and frequently accompanied by postural tremor, which can be elicited during arm extension. In many cases, the kinetic component exhibits greater amplitude than the postural one, and an intention tremor is also present in a significant proportion of patients. Over time, the tremor amplitude tends to increase, indicating the progressive course of the condition. While upper limb tremor is the most common manifestation, tremor may also affect the head, neck, jaw, and legs, often developing after several years of disease progression. Some individuals may exhibit rest tremor without accompanying parkinsonian features [68]. Notably, ET may evolve into Parkinson’s disease, with an estimated four- to fivefold increased risk [71]. Non-motor features include mild cognitive deficits, anxiety, depressive symptoms, personality changes, and sensory abnormalities such as hearing and olfactory dysfunction. The expanded category of ET plus includes patients who meet criteria for ET but also exhibit additional soft neurological features such as impaired tandem gait, questionable dystonia, or cognitive complaints [5]. Although this category aims to better characterise the spectrum of the disorder, it has also generated debate due to its vagueness and potential overlap with other tremor syndromes [38]. A recent study by Tsuboi et al. found that patients with ET plus tend to have more severe tremor, greater involvement of the head, voice, and lower limbs, and higher motor and non-motor symptom scores compared to those with ET. ET plus was also associated with older age at onset and longer disease duration [72]. Ancillary tools such as accelerometery, electromyography, and artificial intelligence-enhanced video or spiral drawing analysis are being explored to support diagnosis, particularly in distinguishing ET from PD [55].

#### 9.1.5. Diagnosis

The diagnosis of ET is primarily clinical, characterised by the presence of a bilateral upper limb action tremor that persists for at least three years, in the absence of other neurological signs, such as dystonia, ataxia, or parkinsonism. Table 1 summarises the 2018 consensus diagnostic criteria for ET and ET plus [5]. A detailed medical history is crucial for ensuring diagnostic precision. Patients commonly report difficulties with fine motor activities such as writing, handling utensils, or using electronic devices. A positive family history is frequently observed, particularly in cases of early-onset essential tremor, reflecting the high heritability of the condition [73]. Neurological examination typically reveals a rhythmic, oscillatory tremor affecting the upper limbs during posture and voluntary movement. The tremor may intensify during goal-directed actions, reflecting an intentional component, while a resting tremor can occasionally appear in advanced stages, usually confined to the arms. Kinetic tremor, best evaluated with finger-to-nose tasks or Archimedes spiral drawings, is a prominent feature. Unlike dystonic or parkinsonian tremors, the spiral in ET typically displays a well-defined axis and a pattern dominated by wrist flexion and extension movements [9,39,74].

Distinguishing ET from other tremor syndromes is essential, especially in cases with overlapping features. Postural tremor in ET appears early and predominantly affects the wrist, whereas in Parkinson’s disease, it may present after a short delay (re-emergent tremor) and involves pronation–supination of the fingers. Although a resting component can be seen in up to 20% of ET patients, it is more typical of Parkinson’s disease and should prompt evaluation for rigidity or bradykinesia. Cephalic and voice tremors are also observed in ET, typically with rhythmic, constant oscillations that can help differentiate it from dystonic tremors or spasmodic dysphonia [39]. Misdiagnosis is not uncommon, especially in early or atypical presentations, and studies have shown a significant proportion of patients diagnosed with ET may later be reclassified [38].

Electrophysiological studies and imaging are not routinely required but may support the diagnosis in complex cases. For instance, tremor frequency measured by accelerometery or EMG, or the presence of synchronous agonist–antagonist muscle activity, may aid in distinguishing ET from Parkinson’s disease or dystonia. Additionally, metabolic causes and medication-induced tremor must be ruled out when clinically indicated [10,39,74]. Ultimately, diagnosis relies on clinical expertise in recognising the core features and excluding alternative explanations through a systematic evaluation.

#### 9.1.6. Treatment

The treatment of ET is primarily symptomatic and pharmacological in nature. Propranolol (the only FDA-approved medication for ET), a non-selective beta-adrenergic blocker, and primidone, an antiepileptic barbiturate, remain the first-line therapies due to their consistent efficacy in reducing tremor amplitude, particularly in the upper limbs [55,56]. Both agents are modestly effective, reducing tremor amplitude by 50% to 70% in a significant proportion of patients, though long-term adherence is low due to limited efficacy and side effects [75,76]. More than half of patients discontinue initial therapy, and a significant proportion cycle through multiple medications [77]. Beta-blockers such as atenolol and metoprolol may be used in patients with contraindications to propranolol [74]. Primidone side effects, like ataxia and sedation, can be mitigated through gradual titration or pretreatment with phenobarbital [78]. Although other medications such as topiramate, gabapentin, alprazolam, and phenobarbital have demonstrated some efficacy, their results are less consistent [79,80]. In cases of refractory ET, particularly with head or voice tremors, botulinum toxin type A injections may be beneficial [81,82]. Various other pharmacological agents, including T-type calcium channel antagonists (e.g., ulixacaltamide, suvecaltamide), modulators of GABAA (e.g., SAGE-324) and GABAB receptors, benzodiazepines, glutamatergic modulators, and drugs that reduce LINGO-1 expression, have been explored, often with variable or limited success [55,83]. Table 2 summarises the pharmacological treatments for ET, classified according to the American Academy of Neurology’s levels of recommendation [56].

Nonpharmacological strategies are considered in mild cases and include occupational therapy to optimise task performance, adaptive tools such as weighted utensils, and wearable orthotic devices that stabilise the limb through mechanical or gyroscopic means [74].

For patients with disabling tremor refractory to pharmacological therapy, surgical interventions like deep brain stimulation (DBS) or MRI-guided focused ultrasound (MRgFUS) offer effective symptom control. DBS remains the most widely used modality due to its reversibility and capacity for bilateral treatment, while MRgFUS provides a non-invasive alternative with comparable efficacy; however, long-term outcomes remain under investigation [83,84,85].

Additional techniques, including peripheral limb cooling and peripheral nerve stimulation, have shown potential as therapeutic options. Peripheral nerve stimulation—also referred to as transcutaneous afferent patterned stimulation—involves the application of patterned electrical stimulation to peripheral nerves with the aim of modulating central tremor pathways. This approach has recently received regulatory approval for clinical use [74,83,86].

Despite ongoing research into novel treatments, no alternative has surpassed the effectiveness of propranolol and primidone to date.

### 9.2. Enhanced Physiological Tremor

The amplification of normal physiological tremor to a more visible level characterises enhanced physiological tremor (EPT). It typically oscillates at 8 to 12 Hz and may be accentuated by reversible factors such as anxiety, fatigue, hyperthyroidism, and certain medications. Clinically, it often presents as a bilateral, symmetrical action tremor involving both postural and isometric components, and may occasionally be observed at rest [87].

EPT exhibits a tremor frequency significantly higher than that of essential or parkinsonian tremor, which provides a key differentiating feature [88]. Burst durations observed on electromyography are notably short (<50 ms), and a synchronous pattern of antagonist muscle activation is commonly observed [89]. Frequency variability is another important marker. EPT shows significantly greater frequency spread compared to essential or parkinsonian tremor, and this instability is thought to reflect a peripheral or reflex component rather than a central tremor generator [88,89].

Task-related features are particularly informative in diagnosing EPT. Loading the limb with a 500–1000 g weight often results in a notable reduction in tremor frequency, observed in 42% of patients with EPT, whereas such a response is rare in other tremor types [90]. Unlike parkinsonian tremor, EPT does not show an amplitude increase during mental tasks. Similarly, intermuscular coherence in EPT is typically lower than in essential or parkinsonian tremor, suggesting weaker coupling between muscles during tremor generation [91].

Diagnostic strategies that combine multiple features have demonstrated high accuracy. A tool using the presence of high frequency, frequency variability greater than 1.75 Hz, and frequency reduction with loading demonstrated 84% sensitivity and 94% specificity for identifying EPT among patients with upper limb tremor [88].

Although EPT is benign, neurophysiological assessments can aid in its differentiation from pathological tremor syndromes.

### 9.3. Focal Tremors

Primary focal tremors predominantly affect specific body regions, most commonly the craniocervical area, including isolated tremors of the head, voice, or buccolingual regions, such as the lower face, jaw, and tongue. These tremors require careful diagnosis to exclude conditions like cerebellar or brainstem pathologies, parkinsonian syndromes, Wilson’s disease, or tremors caused by toxins or medications. Over time, some patients initially diagnosed with primary focal tremor may develop milder hand tremors and be reclassified as having essential tremor or dystonia if sustained movements become evident [5].

#### 9.3.1. Vocal Tremor

Vocal tremor (VT) is a laryngeal movement disorder characterised by rhythmic, involuntary oscillations of the laryngeal muscles at frequencies typically between 4 and 11 Hz. These oscillations occur during sustained vowels or spontaneous speech and are not mitigated by singing or using falsetto. VT may extend to extralaryngeal structures such as the tongue base, soft palate, pharyngeal walls, mandible, and limbs. While tremor direction is usually horizontal or mixed, vertical tremor is rare [92]. VT most commonly appears in association with essential tremor, Parkinson’s disease, and spasmodic dysphonia (SD), but it can also present in isolation or other systemic conditions [93].

VT predominantly affects older adults and shows a marked female predominance (92.9%) [92]. Most cases are associated with broader neurological syndromes, with ET being the most frequent comorbidity (57.1%), followed by PD (14.3%); only 28.5% of cases are considered focal. Although rest tremor is common, many patients also exhibit postural and kinetic components of tremor. In PD, vocal impairment does not consistently align with the severity of general motor symptoms, suggesting distinct neural mechanisms. In ET, voice tremor affects 18–30% of patients and typically manifests as a regular, symmetric kinetic tremor. In contrast, SD-related tremor arises from dystonic co-contractions and is irregular, absent at rest, and often linked to abnormalities in sensorimotor circuits [93].

Diagnosis requires input from otolaryngology, neurology, and speech pathology. Tools such as flexible laryngoscopy, acoustic analysis, voice perceptual profiles, and electromyography are used to characterise the features of the tremor [93]. The underlying disorder guides treatment. Dopaminergic medications in PD and beta-blockers or primidone in ET may offer limited benefit. Botulinum toxin injections remain a mainstay for both ET-related and dystonic VT, tailored to tremor direction and location. In refractory cases, deep brain stimulation or focused ultrasound thalamotomy may be considered [94,95,96].

#### 9.3.2. Head Tremor

Head tremor (HT) is a clinically heterogeneous condition observed across several movement disorders, including essential tremor, cervical dystonia (CD), and Parkinson’s disease, and it may also present as an isolated phenomenon. The epidemiology and clinical features of HT vary depending on the underlying disorder; accurate diagnosis is essential for effective management.

Isolated head tremor (IHT), defined as head tremor without dystonic postures or tremor in other body regions, has traditionally been associated with ET. However, recent evidence suggests that IHT may more closely resemble a dystonic phenotype. Although familial aggregation and early onset in first-degree relatives of ET patients support a possible link to ET, clinical features such as irregular tremor, lack of response to ET-targeted medications, and beneficial response to botulinum toxin support a dystonic mechanism. Neurophysiological studies further support this view, revealing abnormalities in somatosensory temporal discrimination and blink reflex recovery curves in IHT patients—markers typically associated with dystonia [97].

In ET, HT often co-occurs with hand tremor and may present with rhythmic “yes–yes” or “no–no” oscillations. It tends to be more frequent in women and individuals with longer disease duration, suggesting both a phenotype and a state-dependent feature. Neuroimaging studies in ET patients with HT have shown cerebellar involvement, and speech analysis studies indicate more pronounced cerebellar dysfunction in ET patients with HT compared to those without it or to CD patients with HT. This includes altered pitch, slowed articulation, and greater acoustic instability, reflecting impaired cerebellar timing [98].

HT is also a common feature in CD, where it may be the presenting symptom. It is frequently associated with subtle dystonic postures, null-point phenomena, or sensory tricks. Neuroimaging findings suggest cerebellar vermian atrophy in CD patients with HT, distinguishing it from ET. Additionally, task-induced changes in head tremor may further assist in distinguishing between these two conditions. In CD, HT often persists in the supine position and is typically more irregular in pattern. Treatment responses vary, but botulinum toxin remains the most effective therapy [3,99].

In PD, HT is less frequently reported, occurring in approximately 8.3% of patients. It tends to emerge later in the disease course, particularly in those with severe tremor-dominant phenotypes. While HT in PD often responds well to dopaminergic therapy or subthalamic nucleus deep brain stimulation, its response is inconsistent in cases with comorbid ET or CD [100].

#### 9.3.3. Palatal Tremor

Palatal tremor (PT) is a rare movement disorder characterised by involuntary, rhythmic contractions of the soft palate. It is subclassified into essential palatal tremor (ePT), symptomatic palatal tremor (sPT), and progrggessive ataxia with palatal tremor (PAPT), each differing in aetiology, clinical presentation, and prognosis [101].

ePT is idiopathic and typically affects younger individuals with equal sex distribution [102]. It involves contraction of the tensor veli palatini muscle, innervated by the trigeminal nerve (cranial nerve V). It may originate from central mechanisms, such as hyperactivity in the brainstem or inferior olive, as observed on functional MRI, or from peripheral triggers, such as upper respiratory infections or nasal inflammation. ePT often presents with an audible ear click, and it may persist during sleep in about half of the cases. Some patients exhibit voluntary or functional features, including distractibility and entrainment [103,104].

In contrast, sPT occurs predominantly in older males and results from lesions in the dentato-rubro-olivary pathway (Guillain–Mollaret triangle). This leads to hypertrophic olivary degeneration (HOD) and involves the contraction of the levator veli palatini muscle, which is innervated by cranial nerves IX and X. Symptoms may extend to the larynx, pharynx, and other branchial arch derivatives [102]. Clinical features include ataxia, ocular tremor, Holmes tremor, dysarthria, and pendular nystagmus. MRI typically reveals HOD in the medulla, which can be unilateral or bilateral depending on the lesion’s location [101].

PAPT is marked by progressive cerebellar ataxia with concurrent palatal tremor and may be sporadic or familial. Familial forms are associated with genetic mutations such as POLG, SCA20, or Alexander disease. Recent postmortem studies have suggested a neurodegenerative basis with tau pathology, pointing to a potential overlap with tauopathies [101].

The diagnosis of PT relies on clinical examination supported by neuroimaging, especially MRI, to identify HOD. Laboratory tests and genetic panels may aid in identifying underlying causes in sPT or PAPT. Treatment remains empirical; ePT may respond to agents such as clonazepam, valproate, carbamazepine, phenytoin, gabapentin, flunarizine, or lamotrigine, although responses are variable. Botulinum toxin injections into the affected muscles have shown symptomatic benefit but may cause transient side effects, such as velopharyngeal insufficiency. Management of sPT focuses on addressing the causative lesion, with surgical interventions, such as stereotactic ablation, offering potential benefits in select cases. PAPT lacks effective therapies to halt progression, although symptom relief has been reported with gluten-free diets or chenodeoxycholic acid [101,102].

#### 9.3.4. Unusual Focal Tremors

Hereditary chin tremor (HCT, also known as familial geniospasm) is a rare autosomal dominant condition characterised by episodes of involuntary, oscillatory, rhythmic movements of the chin, primarily affecting the mentalis muscle. Symptoms typically manifest at birth or in early childhood and peak during early adulthood. Although geniospasm tends to lessen with age, complete spontaneous remission is uncommon, occurring in fewer than 10% of cases. The tremor episodes, which last from seconds to hours, may be precipitated by emotional stimuli or anxiety and exhibit variable amplitude and frequency, ranging from 2 to 11 Hz. Neurophysiological studies have suggested that the movements may be better classified as myoclonus due to their fast, jerky, and irregular nature. Though generally benign and not interfering with speech, drinking, or sleep, HCT may cause social embarrassment, sleep disturbance, or tongue injuries. Treatment responses to pharmacological agents, including benzodiazepines and anticonvulsants, are usually poor; however, botulinum toxin injections have demonstrated sustained efficacy in symptom control [105,106].

Rabbit syndrome (oral vertical dyskinesia) is an orofacial tremor typically resulting from prolonged use of antipsychotic drugs, particularly those with strong dopamine D2 receptor antagonism and low anticholinergic activity, such as haloperidol and risperidone. It is characterised by fine, rhythmic, vertical movements (approximately 3–5.5 Hz) of the oral, perinasal, and masticatory muscles, often resembling a rabbit’s chewing motion. The syndrome has a reported prevalence of 2.3–4.4% among patients treated with antipsychotics. The onset is usually insidious, emerging after months or years of treatment, and the movements may persist even after discontinuation of the offending agent. Although it resembles parkinsonian tremor in some respects, rabbit syndrome is distinguished by the absence of limb involvement and a favourable response to anticholinergic medications. In addition to cases linked to neuroleptics, it has also been reported following the use of agents such as imipramine, citalopram, and methylphenidate [105,107,108].

Isolated tongue tremor is an uncommon movement disorder in which rhythmic, alternating protrusion–retraction movements of the tongue, typically at a frequency of 3–5 Hz, occur without involvement of the palate. Although tongue tremor can accompany essential tremor, Parkinson’s disease, or symptomatic palatal tremor, isolated cases have been described in association with brainstem or cerebellar astrocytomas, electrical injury, gamma knife radiosurgery for vestibular schwannoma, and anti-Hu paraneoplastic syndromes. Clinically, the tremor predominantly affects the posterior part of the tongue and may be observed at rest, during tongue protrusion, or in both conditions. The course is often self-limiting, with a reported duration of about six months, and some patients may report symptomatic improvement with agents such as central anticholinergics or sodium valproate [105,109].

### 9.4. Task- or Posture-Specific Tremor

Task-specific tremor is an action tremor that occurs exclusively during the performance of particular motor tasks, most commonly involving the upper limbs. In some cases, this tremor appears only when the patient adopts a specific posture, referred to as posture-specific tremor. The most well-known subtype is primary writing tremor, though similar tremors have been documented in individuals performing precise motor activities. These include orolingual tremor during drinking, chin tremor triggered by toothbrushing, finger tremor reported in carrom players, hand tremor while driving, head tremor in billiards players, and other tremors in musicians and sportspeople [3].

Primary writing tremor (PWT) is a task-specific tremor manifesting exclusively during writing or when assuming a writing posture, and it typically affects the dominant hand. The tremor usually emerges around the sixth decade of life, though earlier onset may occur, and it is generally sporadic, with rare familial cases exhibiting autosomal dominant inheritance. Clinically, PWT presents as a non-progressive 5–7 Hz tremor that may worsen with anxiety or rapid writing and may be temporarily relieved by alcohol. It is categorised into Type A (tremor starts during writing) and Type B (tremor appears upon assuming a writing position). Its diagnosis is often challenging due to clinical overlap with writer’s cramp, though neurophysiological studies show differences, such as preserved reciprocal inhibition of the H-reflex in PWT, in contrast to writer’s cramp [110].

The pathophysiology of PWT remains debated. Some researchers propose that it is a variant of task-specific focal dystonia, while others suggest it is a form of essential tremor, and a third view supports PWT as a distinct clinical entity. Neuroimaging and electrophysiological studies support each of these perspectives. Functional MRI findings reveal both overactivation of primary and supplementary motor areas and underactivation of the cingulum. In contrast to dystonia, intracortical inhibition and spinal excitability are generally preserved in PWT [110]. However, recent observations support its inclusion within the spectrum of dystonic tremors rather than ET. In a recent study, the “writing on the wall manoeuvre”—writing on a vertical surface—led to a marked reduction in tremor in two patients with type A PWT. This improvement is interpreted as evidence of a dystonic mechanism, given the presumed engagement of an alternative motor program during this manoeuvre. The absence of benefit in patients with ET reinforces this interpretation [111].

Currently, no randomised controlled trials exist to guide treatment, and management remains symptomatic. Pharmacological agents, such as propranolol, primidone, and anticholinergics, have yielded inconsistent results, with only partial improvement in many cases. Botulinum toxin injections have shown moderate to marked efficacy in selected patients. Surgical approaches, intense brain stimulation targeting the ventral intermediate nucleus of the thalamus, have demonstrated substantial tremor reduction in cases that are refractory to other treatments. Adaptive devices that redistribute the motor load from distal to proximal muscles have also shown promise as non-invasive alternatives. Further research is essential to clarify the nosological status of PWT and to establish evidence-based therapeutic guidelines [110].

### 9.5. Primary Orthostatic Tremor

Primary orthostatic tremor is a rare and intriguing syndrome characterised by a high-frequency tremor (13–18 Hz) predominantly affecting the lower limbs during quiet standing, leading to unsteadiness and a compelling urge to walk, sit, or lean to alleviate symptoms [112,113]. It typically begins after the age of 50, with a female predominance, and can significantly impact quality of life by restricting the ability to perform standing activities, such as cooking or queuing [113,114]. While most cases are idiopathic, some have been associated with other neurological conditions. The pathophysiology is not fully understood, but involvement of the ponto-cerebello-thalamo-cortical network has been suggested [114].

Clinical signs may be subtle. The tremor is often imperceptible on routine examination but can sometimes be detected by palpation or auscultation of the quadriceps or calves. The “helicopter sign”, an audible correlate of the tremor, and the recently validated “hem sign”, describing the fluttering of a garment hem over the thigh, can aid in clinical recognition. Additional frequent signs include bent knees, a broad base of support while standing, and abnormal tandem gait, although no consistent cerebellar or extrapyramidal findings are observed [115].

Diagnosis relies on clinical history and confirmation by surface EMG, which reveals characteristic rhythmic bursts during stance that disappear upon sitting or walking [112,114]. Despite the progressive perception of the disorder among patients, longitudinal studies suggest that daily functioning and subjective well-being remain relatively stable over time, possibly due to adaptation [113].

Pharmacologic treatment is often unsatisfactory, and commonly used agents such as clonazepam and gabapentin yield partial benefit. Perampanel, an AMPA receptor antagonist, has shown promising results, including complete symptom resolution in isolated cases, but is frequently associated with adverse effects [112,116]. In medication-refractory patients, DBS, particularly targeting the ventral intermediate nucleus of the thalamus or the caudal zona incerta, has been associated with substantial improvements in stance duration and daily functioning. However, long-term efficacy may wane, and side effects such as dysarthria or paraesthesia are common [114,116]. Spinal cord stimulation has been explored in a small number of patients, with variable and limited results [114].

### 9.6. Tremor with Prominent Additional Signs

#### 9.6.1. Tremor in Dystonia

Tremor in dystonia encompasses a heterogeneous group of motor phenomena that may manifest either within a dystonic body region—usually termed dystonic tremor—or in an anatomically distinct area, referred to as tremor associated with dystonia (TAWD). This distinction, however, remains a subject of ongoing debate in both clinical and conceptual contexts [117,118].

Epidemiological data suggest that tremor is common in dystonia, with prevalence estimates ranging from 10% to over 50%, depending on the clinical context and diagnostic criteria used [118,119]. Demographic and clinical profiles further support their differentiation. For instance, dystonic tremor tends to occur in younger patients with isolated dystonia, while TAWD often appears in older individuals and may be associated with essential tremor or Parkinson’s disease [92].

Pathophysiologically, tremor in dystonia is thought to result from abnormal interactions within the basal ganglia-thalamo-cortical and cerebello-thalamo-cortical circuits, with the pattern of neural activity differing between jerky, irregular dystonic tremors and more sinusoidal, regular tremors that resemble essential tremor [117,119]. These divergent mechanisms have led some experts to propose replacing ambiguous terms like dystonic tremor with more descriptive labels such as jerky dystonia or dystonia with tremor [118].

Clinically, dystonic tremor is often irregular, asymmetric, and influenced by posture, sometimes exhibiting a *null point* or being suppressible by a *sensory trick*. In contrast, TAWD may mimic essential tremor, complicating diagnosis [119,120]. Diagnostic accuracy is further challenged by the subjective interpretation of features such as jerkiness and the presence of mild dystonia, leading to poor inter-rater agreement [118]. Electrophysiological tools, such as surface EMG, accelerometery, and motion analysis, have been proposed to enhance diagnostic precision by quantifying rhythmicity, frequency, and waveform shape [117].

Treatment strategies should be individualised. Botulinum toxin is often effective for focal dystonic tremor, whereas TAWD may respond better to medications like propranolol or primidone. Invasive interventions, including DBS, require careful selection of surgical targets, as stimulating the thalamus may worsen coexisting dystonia [92,119]. Ultimately, the need for revised terminology and objective diagnostic criteria remains central to optimising care and advancing research [118,120].

#### 9.6.2. Tremor Related to Parkinsonism

Tremor associated with parkinsonism encompasses a heterogeneous group of motor manifestations observed in Parkinson’s disease and atypical parkinsonian syndromes such as multiple system atrophy (MSA), progressive supranuclear palsy (PSP), and corticobasal degeneration (CBD). In PD, tremor typically presents as a rest tremor with a frequency of 4–6 Hz, often beginning unilaterally and exhibiting a characteristic “pill-rolling” pattern. A distinct subtype is re-emergent tremor, a postural tremor that appears after a latency when the arms are held outstretched; it shares the same frequency as rest tremor and is often considered a manifestation of the same underlying generator [3,121]. Postural and kinetic tremors are also frequently observed in PD and may resemble essential tremor, particularly in tremor-dominant phenotypes, which complicates the differential diagnosis [3,121]. Kinetic tremor often coexists with rest and postural tremors in PD and may become more prominent as the disease progresses [122].

Pathophysiologically, PD tremor arises from complex interactions between the basal ganglia and cerebello-thalamo-cortical circuits. The “dimmer-switch” model posits that the basal ganglia act as a trigger, while the cerebellar loop modulates tremor amplitude [123]. Recent evidence suggests a more active role of the primary motor cortex (M1) in tremor genesis and suppression, indicating that M1 may serve as a key regulatory node within the tremor network [124]. Computational models further support the view that tremor results from unstable feedback in a compromised motor control system, driven by excessive thalamic inhibition and mismatches between internal models and sensory feedback [125].

Clinically, tremor may be the predominant feature in some PD phenotypes, while in others it may be mild or absent. In atypical parkinsonism, tremors are less frequent and often differ in quality. For instance, MSA may present with jerky, myoclonic tremor, and PSP typically shows minimal tremor, whereas CBD may exhibit dystonic or irregular tremor patterns [3,121].

Levodopa remains the first-line treatment for parkinsonian tremor, though its efficacy is variable. A significant proportion of patients experience tremor that is only partially or non-responsive to dopaminergic therapy, underscoring the contribution of non-dopaminergic mechanisms. Alternative pharmacologic options include anticholinergics, beta-blockers, and clozapine, although these are limited by tolerability and side effects. For medication-resistant tremor, DBS, particularly targeting the VIM or STN, remains the most effective intervention [122].

In addition to these traditional DBS targets, the zona incerta (ZI) has emerged as a promising alternative site for neuromodulation. ZI-DBS has demonstrated efficacy in reducing rest, postural, and action tremors, with some studies reporting more robust and sustained tremor control than VIM-DBS in certain patients [35,126].

The therapeutic benefits of ZI stimulation may stem from its broad functional connectivity and its capacity to modulate tremor-related network oscillations. ZI-DBS may be particularly useful in patients with atypical or mixed tremor phenotypes, poor response to conventional DBS targets, or significant postural instability [36]. Ongoing clinical and imaging studies are expected to refine the definition of optimal patient selection and stimulation parameters.

#### 9.6.3. Cerebellar Tremor

Cerebellar tremor is a low-frequency (<5 Hz) intention tremor typically observed during visually guided, goal-directed movements and is most prominent as the limb approaches its target. It typically affects the upper limbs but can involve the head, voice, trunk, and lower limbs. It is commonly associated with structural lesions of the cerebellum or its efferent pathways. It is often accompanied by other signs of cerebellar dysfunction, such as dysmetria (inaccurate movement control), ataxia (lack of coordination), hypotonia (reduced muscle tone), or nystagmus (involuntary rhythmic eye movements) [3,105].

Aetiologically, cerebellar tremor arises from a range of underlying conditions, including multiple sclerosis, stroke, tumours, trauma, and genetic ataxias. It may also be observed in degenerative disorders, such as FXTAS and certain spinocerebellar ataxias, where the tremor can precede or dominate the clinical presentation [3].

From a pathophysiological perspective, two primary mechanisms have been proposed. One involves dysfunction of the cerebellar forward model, which typically predicts the sensory consequences of movement. Damage to this system results in impaired error correction, leading to oscillatory movements characteristic of cerebellar tremor. The other mechanism, involving the inferior olive, postulates that synchronised subthreshold oscillations in olivary neurons—transmitted through climbing fibres—can generate rhythmic activity in Purkinje cells, thereby contributing to tremor even in the absence of goal-directed movement [127].

Clinically, cerebellar tremor is distinguished by its worsening during voluntary action and is rarely observed at rest. Diagnosis is primarily clinical, supported by the presence of accompanying cerebellar signs and neuroimaging findings that identify lesions in areas such as the dentate nucleus or superior cerebellar peduncle [105]. Pharmacological treatments for cerebellar tremor, such as clonazepam, isoniazid, and topiramate, offer modest benefits [128,129]. In severe cases, surgical targeting of the ventral intermediate nucleus or the posterior subthalamic area (PSA) may reduce tremor amplitude; however, outcomes depend on the severity of tremor and coexisting neurological deficits, making a careful risk–benefit evaluation necessary. DBS targeting these regions has also been explored, but the results remain inconsistent, and further research is warranted to clarify its therapeutic potential [3,130].

#### 9.6.4. Holmes Tremor

Holmes tremor is a rare and complex tremor syndrome characterised by a combination of rest, postural, and kinetic tremors of low frequency (2–5 Hz) and high amplitude, typically affecting a single upper limb [3,131]. Although infrequent, Holmes tremor significantly impairs quality of life and functional independence due to its debilitating and persistent nature [132].

Aetiologically, Holmes tremor arises secondary to structural brain lesions affecting the midbrain, thalamus, cerebellum, or brainstem, most commonly due to stroke, trauma, demyelination, tumours, or infections. The onset of symptoms is typically delayed, occurring weeks to months after the initial insult [3,131,132]. Genetic findings, including mutations in *PNPLA6*, have also been linked to Holmes tremor in isolated cases, suggesting a possible hereditary contribution [131].

The pathophysiology of Holmes tremor is attributed to the disruption of both the dopaminergic nigrostriatal system and the cerebellothalamic or dentato-rubro-thalamic circuits, with recent lesion network mapping studies implicating a wider motor network. Neuroimaging typically reveals lesions affecting these pathways, and functional imaging may reveal dopaminergic deficits, which aid in the diagnostic process [3,131].

Management remains challenging, as pharmacological treatments often yield inconsistent results. Levodopa has shown partial benefit, particularly for the resting component, while dopamine agonists and anticholinergics have been effective in selected cases. Other agents, including beta-blockers and anti-seizure drugs, generally show poor efficacy [133]. Surgical interventions are increasingly employed in refractory cases. DBS targeting the VIM, GPi, or STN has provided substantial symptom relief in many patients [131,133]. More recently, MRI-guided laser interstitial thermal therapy (MRIgLITT) thalamotomy has emerged as a promising lesion-based approach, demonstrating significant tremor reduction in a small series. However, improvements in fine motor function and quality of life remain modest, and one patient experienced severe but transient adverse effects [132].

#### 9.6.5. Myorhythmia

Myorhythmia is a rare hyperkinetic movement disorder characterised by slow (1–4 Hz), rhythmic, often jerky oscillations involving craniofacial and limb muscles. These movements can occur at rest or during posture and are typically abolished during sleep. Although considered uncommon, its recognition is clinically relevant due to its frequent association with structural brain lesions and potentially treatable aetiologies [3,134]. Its classification has been subject to debate; however, the 2018 consensus recognised it as a form of tremor [5].

Atiologically, myorhythmia is linked to a wide range of conditions, most notably infectious (such as Whipple’s disease), autoimmune (including anti-NMDA receptor encephalitis and Hashimoto’s encephalopathy), vascular lesions affecting the brainstem or thalamus, and paraneoplastic or drug-induced causes. Oculo-masticatory myorhythmia is pathognomonic of Whipple’s disease [3,134]. The disorder is often associated with lesions involving the Guillain–Mollaret triangle, particularly in patients with brainstem pathology or demyelinating diseases such as multiple sclerosis [135].

From a pathophysiological perspective, myorhythmia has been hypothesised to result from rhythmic discharges originating in subcortical or brainstem pacemaker structures. The study by Elkhooly and colleagues provided the first quantitative analysis of its synchrony and rhythmicity, demonstrating highly rhythmic 2.5–3.2 Hz oscillations in a patient with multiple sclerosis. Using gyroscopic sensors, the authors observed narrow spectral bandwidths and strong coherence between the head and upper limbs, confirming the tremor-like nature of myorhythmia despite its clinical appearance as jerky or arrhythmic [135].

Clinically, myorhythmia must be distinguished from other slow movement disorders such as Holmes tremor and palatal tremor. Diagnosis is primarily clinical but may be aided by electrophysiological recordings and imaging studies. Neurophysiologically, myorhythmia presents with semi-rhythmic discharges lasting 200–300 ms in EMG, longer than those observed in myoclonus but shorter than classic tremor discharges. The recent use of inertial sensors has enabled a more precise characterisation of its rhythmic and synchronous features [134,135].

Treatment is directed toward the underlying cause when it is identifiable. Antibiotics are indicated in infectious cases such as Whipple’s disease, while immunotherapy may benefit autoimmune or paraneoplastic aetiologies. Symptomatic pharmacological treatment is often unsatisfactory, although some benefit has been reported with tetrabenazine, gabapentin, or botulinum toxin in selected cases [134].

### 9.7. Other Forms of Tremor

#### 9.7.1. Functional Tremor

Functional tremor (FT, formerly psychogenic) is the most prevalent phenotype of functional movement disorder, representing 40–50% of cases in specialist clinics. It predominantly affects women and typically begins in young or middle adulthood, though it can also occur in paediatric and elderly populations. Risk factors include psychosocial stressors and comorbid functional symptoms, such as chronic pain or non-epileptic seizures [41,136].

Pathophysiologically, FT is associated with altered connectivity between limbic and motor regions, impaired emotion regulation, excessive attentional focus on the affected body part, and a disrupted sense of agency [41,136]. Neuroimaging studies reveal abnormal activation of the temporoparietal junction and supplementary motor area, as well as increased amygdala reactivity, consistent with the predictive processing model of brain function [136]. This model posits that strong internal predictions override sensory inputs, leading to involuntary motor symptoms experienced as uncontrollable [137].

Clinically, FT is characterised by tremor of variable frequency and amplitude that may affect multiple body parts, most commonly the limbs. It often presents with a combination of rest, postural, and kinetic components. Hallmark features include distractibility (reduction during distraction), entrainment (synchronisation with voluntary rhythmic movement of another limb), suggestibility (modulation by external cues), selectivity (symptoms limited to specific contexts), variability (fluctuation across time and tasks), the coactivation sign (simultaneous antagonist contraction before reemergence), abrupt onset, and non-progressive course [136]. These signs support a rule-in diagnosis, which should rely on positive clinical features rather than exclusion or the notion of incongruence with neurological disease [41,137].

The diagnostic process should emphasise internal inconsistency rather than so-called incongruence, which has been critiqued as an unreliable and outdated construct [137]. Electrophysiological testing, including surface electromyography and accelerometery, can support the diagnosis, particularly when entrainment or distractibility is not readily apparent during the clinical examination [136].

Current treatment strategies for FT prioritise patient education and multidisciplinary interventions. Cognitive behavioural therapy and physiotherapy, which target motor retraining and maladaptive beliefs, have shown promising results. Other emerging approaches include biofeedback, neuromodulation, and digitally delivered therapies; however, further studies are needed to establish their efficacy [41,136].

#### 9.7.2. Neuropathic Tremor

Neuropathic tremor (NT) is a postural and/or kinetic tremor that typically affects the upper limbs and is associated with various peripheral neuropathies. It has been reported in chronic inflammatory demyelinating polyneuropathy (CIDP), paraproteinemic neuropathies, hereditary conditions such as Charcot–Marie–Tooth disease, and more recently, in the recovery phase of Guillain–Barré Syndrome (GBS) [3,138]. The prevalence of tremor in CIDP varies widely, ranging from 3.9% to 58% [139].

The aetiology of NT appears to be multifactorial and varies depending on the underlying neuropathy. While traditionally linked to chronic demyelinating or hereditary conditions, Rajan et al. demonstrated that NT can emerge after GBS, a typically acute immune-mediated neuropathy [138]. Genetic forms such as Charcot–Marie–Tooth disease may present with NT early in the disease course [140].

The pathophysiological basis of NT is not fully understood. However, evidence suggests a central mechanism, likely involving cerebellar dysfunction driven by impaired peripheral feedback. Intraoperative recordings during DBS procedures in patients with IgM paraproteinemic neuropathy revealed synchronised activity between the motor cortex, thalamus, and muscles, supporting the presence of a central oscillator [141]. Similarly, Rajan et al. found electrophysiological features consistent with a cerebellum-driven central oscillator in patients with GBS-associated NT [138].

Clinically, NT is characterised by a fine to jerky postural tremor, often symmetrical and involving the upper limbs. In GBS-associated cases, the tremor typically manifests after motor recovery and may be accompanied by dystonic posturing and overflow movements [138]. In some cases, NT may resemble essential tremor or other tremor syndromes, posing diagnostic challenges [3].

Diagnosis relies on clinical assessment supported by electrophysiological studies to confirm the underlying neuropathy. Tri-axial accelerometery and surface electromyography can help differentiate NT from other tremor types by revealing the presence of central oscillators and characterising frequency and response to loading [138].

Treatment options for NT are limited. Pharmacologic agents, such as propranolol or primidone, may provide partial benefit; however, many cases are refractory [3]. In selected patients, DBS targeting the ventral intermediate nucleus of the thalamus has demonstrated durable efficacy in controlling tremor severity and improving quality of life [139,140,142]. In cases associated with active inflammatory neuropathy, immunotherapy may help manage both the neuropathy and tremor symptoms [139].

#### 9.7.3. Toxin- or Drug-Induced Tremor

Toxin- or drug-induced tremor is a frequent yet often under-recognised form of secondary tremor, typically arising from exposure to pharmacologic agents or substances of abuse. Epidemiologically, it is more prevalent in older individuals, males, and those undergoing polypharmacy, particularly when high doses or immediate-release formulations are used [143]. A broad spectrum of agents is implicated, including antidepressants, lithium, valproate, antiepileptics, bronchodilators, antiarrhythmics, immunosuppressants, chemotherapeutics, and neuroleptics, as well as recreational substances and environmental toxins such as lead, mercury, and manganese [143,144,145].

The pathophysiological mechanisms vary depending on the agent. Tremor can result from enhanced physiological tremor due to peripheral β-adrenergic stimulation, cerebellar or basal ganglia toxicity, or disruption of dopaminergic and serotonergic neurotransmission. For example, valproate and lithium are thought to modulate central oscillatory circuits, while toxins such as manganese accumulate in the basal ganglia, leading to parkinsonism-like symptoms [144,145].

Clinically, the tremor may mimic essential or parkinsonian tremor, with postural and kinetic tremors being most common. However, rest tremor and tremors involving less typical body regions may also occur. Features such as symmetry, a non-progressive course, and the absence of structural findings support a drug-induced origin [3,143]. Diagnosis requires a detailed review of the patient’s medication history and consideration of the temporal relationship between medication use and symptom onset. The Naranjo scale (an adverse drug reaction probability scale) may assist in determining causality [143].

Management primarily involves withdrawal or dose reduction in the offending agent. Symptomatic treatment with β-blockers or primidone may be considered. Although symptoms often remit after discontinuation, some patients may develop persistent or tardive tremor [143].

#### 9.7.4. Other Unusual Tremors

Rare or unusual tremors encompass a diverse group of disorders that are frequently misdiagnosed due to their low prevalence and overlapping features with more common tremor syndromes. While precise epidemiological data are limited, these tremors are increasingly acknowledged as distinct entities within the broader tremor classification framework [3,105].

Among the genetic causes, fragile X-associated tremor/ataxia syndrome and spinocerebellar ataxia type 12 (SCA12) are notable. FXTAS, associated with premutation expansions in the FMR1 gene, typically presents with intention or postural tremor preceding ataxia and cognitive impairment, along with characteristic T2 hyperintensities in the middle cerebellar peduncles. SCA12, caused by CAG repeat expansions, often begins with postural tremor and is especially prevalent in individuals of North Indian ancestry. Autosomal recessive ataxias, such as Friedreich’s ataxia, may also feature tremor, though less commonly [105,146].

Metabolic and toxic causes include Wilson’s disease, in which tremor may manifest as wing-beating or rubral tremor, and tardive tremor, a high-amplitude, low-frequency tremor that arises after prolonged use of dopamine receptor-blocking agents and may persist even after discontinuation of the offending drug [146].

Slow orthostatic tremor, typically occurring at frequencies below 12 Hz, is observed upon standing and may be idiopathic or secondary to structural or autoimmune conditions. It differs from classical orthostatic tremor and can mimic Parkinsonian tremor, complicating diagnosis [105,146]. In multiple sclerosis, tremor—usually of intention or postural type—can be disabling and reflects involvement of cerebellar or brainstem pathways [3].

Paroxysmal or episodic tremors include limb-shaking transient ischemic attacks (TIAs), characterised by brief, irregular tremulous movements due to cerebral hypoperfusion in the context of carotid artery disease, and benign hereditary fidgety shaking disorder, a rare familial episodic tremor syndrome reported in the literature [146]. Finally, tremor has also been reported in Klinefelter syndrome, though its pathophysiology remains unclear [3].

Accurate diagnosis of these rare tremors requires thorough clinical evaluation, often supported by neuroimaging, genetic testing, or assessment of metabolic and autoimmune markers. Management strategies vary widely depending on the underlying cause, ranging from symptomatic treatment to disease-specific interventions such as chelation therapy in Wilson’s disease or revascularisation procedures in limb-shaking TIAs.

### 9.8. Indeterminate Tremor Syndrome

Indeterminate tremor syndrome refers to cases that do not fulfil diagnostic criteria for essential tremor, ET plus or other well-defined tremor syndromes [5]. The term was introduced to address the need for diagnostic clarity in patients whose tremor features are insufficiently specific or atypical. These individuals often exhibit isolated or combined tremors of uncertain significance that cannot be confidently categorised, even after thorough clinical assessment. The syndrome may represent an early or *forme fruste* presentation of an evolving tremor disorder, or a stable, non-progressive condition. In some cases, longitudinal observation reveals the later emergence of diagnostic features consistent with a recognised tremor syndrome, whereas in others, the tremor remains unchanged and remains diagnostically inconclusive [6].

This classification enables clinicians to acknowledge diagnostic uncertainty in patients who do not meet the criteria for established syndromes, thereby promoting careful longitudinal follow-up. It facilitates appropriate clinical management while avoiding premature labelling and supports more transparent communication with patients. Additionally, it encourages research into atypical tremor presentations and ensures more accurate phenotyping in clinical studies.

### 9.9. Paediatric Tremor Syndromes

Tremor can manifest across all paediatric age groups, including the neonatal period. Tremor in neonates is often described using overlapping terms, such as jitteriness or shuddering, and is usually benign, presenting with high-frequency and low-amplitude movements that typically resolve by the third day of life. However, persistent, coarse tremors or those appearing after the third day warrant further evaluation for metabolic disturbances or intracranial pathology. Pathological forms may include neonatal hyperexcitability syndrome or congenital tremors associated with genetic mutations such as those in *MYBPC1*, *CACNA1D*, or *SCN4A* genes.

During infancy, distinct tremor syndromes emerge. Shuddering attacks are brief, high-frequency tremors that resemble shivers without altered consciousness, typically resolving spontaneously by the age of seven. Spasmus nutans presents with head tremors, nystagmus, and head tilt, often idiopathic but occasionally associated with intracranial pathology. Head nodding and bobble-head doll syndrome differ in frequency and amplitude, with the latter characterised by rhythmic, repetitive head movements—typically in a “yes–yes” direction—associated with third ventricular cysts or hydrocephalus and responsive to surgical treatment. Infantile tremor syndrome, primarily seen in the Indian subcontinent, results from vitamin B12 deficiency and manifests with tremor, developmental regression, and hypotonia, showing partial improvement with supplementation.

In older children and adolescents, tremor becomes more common and varied. Essential tremor is the most frequently reported tremor type in this age group, often familial, with onset around six years of age. Although treatment is often unnecessary in childhood, pharmacologic interventions such as propranolol or primidone are considered when significant disability is present. Other causes of tremors in this age group include enhanced physiologic tremor, substance-induced tremors, and functional tremors. The clinical evaluation of paediatric tremor requires careful observation, consideration of associated features, and tailored diagnostic workup based on age and presentation [57].

## 10. Diagnostic Utility of Clinical Neurophysiology in Tremor Syndromes

Clinical neurophysiology plays a crucial role in the diagnostic evaluation of tremor syndromes, providing objective parameters that complement and refine clinical assessments. Standard techniques involve surface EMG and accelerometery to assess tremor frequency, amplitude, variability, and muscle activation patterns during rest, posture, and action tasks [147,148]. These methods are beneficial in differentiating functional tremors. Functional tremors are typically characterised by frequency variability, distractibility, entrainment, and abnormal coherence patterns, while essential tremor and parkinsonian tremor tend to exhibit more stable and syndrome-specific features [10,149]. The use of task-based paradigms and coherence analysis further enhances diagnostic specificity [89]. Differentiating tremor from myoclonus relies on the identification of brief, non-rhythmic bursts on EMG and jerky movements on accelerometery, in contrast to the rhythmic, sinusoidal pattern of tremor. In Parkinson’s disease, tremor typically has a resting component with a characteristic re-emergent pattern during posture, while tremor in atypical parkinsonian syndromes is often less rhythmic and more irregular. Dystonic tremor can be distinguished from essential tremor by its irregular frequency, association with dystonic posturing, and the presence of null points or tremor variability. Essential tremor is generally more rhythmic and stable, with bilateral postural and kinetic components [10].

Neurophysiological studies not only clarify complex or overlapping tremor presentations but also allow quantitative comparison of tremor subtypes. For instance, Tsuboi et al. demonstrated that patients with essential tremor plus exhibit lower tremor frequencies and higher amplitudes than those with essential tremor, although both share similar rhythmicity and activation patterns [72].

Importantly, multiple studies have confirmed the high diagnostic yield of electrophysiological testing, particularly in cases of indeterminate or mixed tremor [10,149]. Overall, clinical neurophysiology offers reproducible and quantitative markers that enhance diagnostic precision when used in conjunction with clinical expertise [147,148].

## 11. Advancing Frontiers in Tremor Diagnosis and Therapy

**Figure 4:**
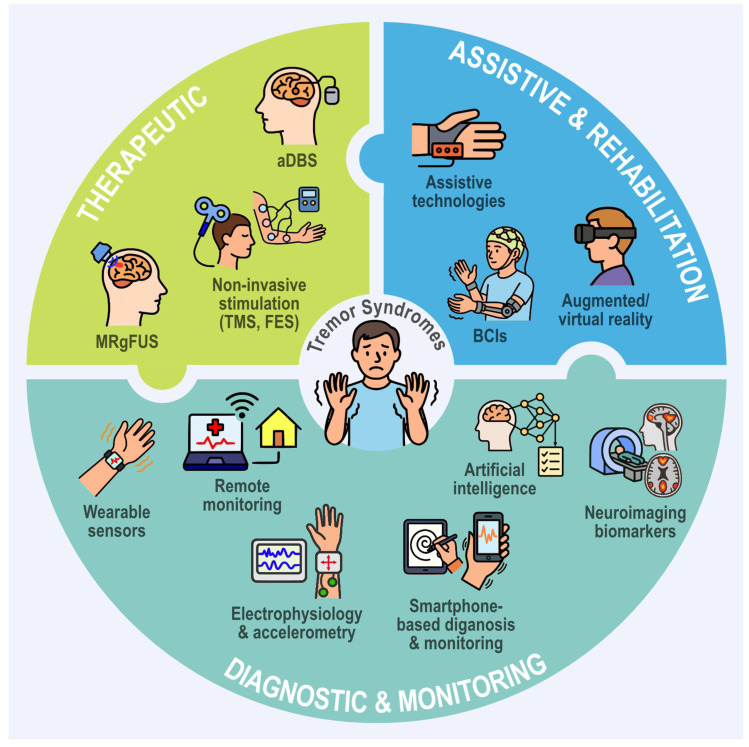
Technological innovations advancing tremor diagnosis and therapy. Emerging technologies are transforming tremor care by enabling more precise diagnoses, individualised therapies, and enhanced rehabilitation. Innovations are grouped into three main domains: therapeutic—encompassing adaptive deep brain stimulation (aDBS), magnetic resonance-guided focused ultrasound (MRgFUS), and non-invasive stimulation techniques such as transcranial magnetic stimulation (TMS) and functional electrical stimulation (FES); assistive and rehabilitation—including assistive devices, brain–computer interfaces (BCIs), and augmented or virtual reality platforms designed to enhance motor function and user engagement; and diagnostic and monitoring—covering wearable sensors, electrophysiology and accelerometery, smartphone-based tools, artificial intelligence applications, remote monitoring systems, and neuroimaging biomarkers. Together, these technologies support a multidimensional, personalised approach to tremor management in both clinical and home settings.

Recent technological advances are redefining the diagnosis and treatment of tremor, offering promising alternatives and complements to conventional pharmacological and surgical interventions. The integration of artificial intelligence (AI), wearable systems, adaptive neuromodulation, and non-invasive techniques is progressively shaping a more personalised and precise approach to managing tremors (Figure 4).

AI and machine learning are increasingly applied to tremor classification and monitoring, leveraging data from inertial sensors, EMG, or smartphones to support differential diagnosis and symptom quantification. Models trained on accelerometery or handwriting tasks have shown high accuracy in distinguishing Parkinson’s disease, essential tremor, and dystonic tremor, although challenges remain in diagnosing overlapping syndromes and capturing real-world variability [150,151]. Explainable AI frameworks and session-specific neural markers are also being explored to enhance transparency and adaptability in tremor decoding algorithms [152].

Wearable technologies have emerged as promising tools for assessing and intervening in tremors. Inertial measurement units (IMUs), smartwatches, and digitising tablets enable objective, continuous tracking of tremor characteristics, with some devices achieving clinical-grade performance [53,54]. Paredes-Acuña et al. highlighted the utility of wrist-worn IMUs in characterising intention tremor in individuals with multiple sclerosis, demonstrating a good correlation with clinical scores, while also emphasising challenges such as task dependency and signal variability [153]. Additionally, wearable devices integrated with machine learning can support real-time tremor suppression or function as diagnostic aids in both clinical and home environments [154,155]. Assistive technologies, such as gyroscopic utensils, orthotic gloves, and robotic exoskeletons, have demonstrated functional benefits for patients during daily tasks; however, long-term usability and patient adherence remain areas for further investigation [156].

Remote measurement and home monitoring have gained relevance with the expansion of telemedicine. Devices like the Parkinson’s Kinetigraph and consumer smartwatches allow tremor evaluation in naturalistic settings, offering an ecologically valid and scalable solution for tracking disease progression or treatment response [154]. Similarly, computer vision approaches have shown promise as contactless, non-invasive methods for tremor detection and classification, particularly when integrated into telehealth platforms [157].

Adaptive deep brain stimulation (aDBS) represents a significant evolution from traditional continuous stimulation. Studies have demonstrated that aDBS, whether driven by thalamic local field potentials, cortical signals, or movement-related biomarkers, can achieve tremor suppression comparable to open-loop DBS while reducing energy consumption and stimulation-related side effects [158,159,160]. Fully implanted systems, cortically driven control, and machine learning-based classifiers have all contributed to making aDBS a clinically translatable approach, with applications extending to the control of orthostatic tremor and nighttime tremor [161,162,163].

Magnetic resonance-guided focused ultrasound (MRgFUS) has emerged as a non-invasive lesioning technique for ET and tremor-dominant PD. By thermally ablating thalamic targets such as the VIM, MRgFUS provides durable tremor reduction without the need for implanted hardware, offering an attractive alternative for patients ineligible for DBS [164,165,166]. Although bilateral treatments and new targets are under exploration, limitations related to irreversibility and patient selection criteria persist.

Non-invasive stimulation techniques, including transcranial magnetic stimulation (TMS) and peripheral or functional electrical stimulation (FES), are emerging as promising alternatives or complements in the management of tremor-related disorders. In ET, cerebellar repetitive TMS (rTMS) has shown modest benefits in some studies, while others have not found significant effects [167,168]. Phase-locked TMS has demonstrated enhanced tremor suppression by aligning stimulation with ongoing oscillatory activity [169]. In Parkinson’s disease, rTMS targeting motor and cerebellar areas can improve bradykinesia and rigidity more consistently than tremor, although dual-site protocols have shown encouraging results [170,171]. In functional tremor, TMS may also serve as a diagnostic tool, with distractibility during stimulation predicting therapeutic response [172]. On the peripheral side, wearable stimulation devices offer non-invasive tremor suppression by delivering patterned input to nerves or muscles, with some closed-loop devices demonstrating tremor reduction rates of up to 81% [173]. Recent studies have refined FES protocols to optimise stimulation parameters, such as amplitude and pulse count, for wrist tremor in PD. Notably, stimulation at or slightly above the motor threshold produced greater suppression than stimulation at subthreshold levels, although responses varied across participants and tremor intensity levels [174].

Brain–computer interfaces have also been proposed as control mechanisms for tremor suppression systems. A multimodal BCI-driven wearable system, utilising EEG, EMG, and IMUs, has demonstrated the feasibility of distinguishing between voluntary and tremorous movements in real time to activate functional electrical stimulation for tremor attenuation [175]. Similarly, non-invasive peripheral nerve stimulation, such as the Cala Trio system, modulates tremor via patterned afferent input and has shown promising results in clinical trials [176].

Finally, augmented and virtual reality technologies are being integrated into rehabilitation protocols. Optical see-through augmented reality systems have been developed to enhance engagement and motor training in patients with tremors, offering interactive feedback and improving movement precision during task-oriented therapy [177]. These platforms show potential for home-based interventions, especially when combined with wearable motion tracking.

These technological innovations will reshape tremor care through multidimensional strategies that combine accurate assessment, real-time intervention, and user-centred design. Continued interdisciplinary collaboration and clinical validation are crucial for translating these advances into routine practice and achieving individualised, responsive, and effective tremor management.

## 12. Discussion

This narrative review has evaluated the evolution of tremor classification, with particular attention paid to the advancements introduced by the 2018 consensus criteria. The transition from a fragmented approach, where tremor syndromes were classified based on semiology, anatomical origin, or aetiology, to a more cohesive and integrated framework represents a significant improvement in understanding and managing tremor disorders. Notably, essential tremor, once regarded as a simple idiopathic condition, is now recognised as a syndromic entity with diverse aetiologies, prompting a shift towards syndromic rather than purely aetiological diagnostic approaches. Emerging diagnostic tools such as neuroimaging and genetic testing further support this paradigm shift, offering new insights into the mechanisms underlying tremor syndromes.

The 2018 consensus criteria introduced a two-axis classification system that has advanced both research and clinical practice. Axis 1 defines the syndromic diagnosis, integrating tremor semiology, clinical history, associated signs, and ancillary testing, while Axis 2 identifies aetiology—whether acquired, genetic, or idiopathic [5]. This framework enables more precise differentiation between tremor types and supports dynamic reclassification as syndromes evolve. The subdivision of ET into essential tremor and ET plus, along with the introduction of the indeterminate tremor category, further enhances diagnostic flexibility and clinical applicability.

Despite these benefits, the 2018 framework presents limitations. Its reliance on expert consensus and existing studies may not fully capture the latest research or emerging syndromes. Moreover, while the classification enhances diagnostic accuracy, it may underrepresent the lived experience of patients and the functional consequences of tremor on quality of life. Additionally, the advancement of neuroimaging and genetic technologies suggests that current nosological definitions may require periodic revision to remain clinically relevant.

Pathophysiologically, converging evidence implicates the cerebello-thalamo-cortical network as a shared substrate across diverse tremor syndromes [18,19,20]. However, distinct oscillatory patterns and regional dysfunctions contribute to syndrome-specific phenotypes. In Parkinson’s disease, the basal ganglia and cerebellum interact dynamically to generate tremors, whereas in ET, cerebellar dysfunction appears to be central. In dystonic and Holmes tremor, more widespread circuit disruptions are implicated [22,23,30,33]. These findings have prompted the proposal of a third axis of classification based on pathophysiological mechanisms; although promising, such an addition would require further validation and standardisation before it can be adopted clinically [18].

Clinically, diagnostic accuracy remains contingent on comprehensive examination and longitudinal reassessment. While clinical neurophysiology and rating scales provide valuable support, their interpretability is limited by inter-rater variability and the nonlinearity between tremor amplitude and scale scores [45,46]. Advanced techniques—such as EMG coherence analysis, neuroimaging biomarkers, and accelerometery—offer more objective and reproducible data, which are instrumental in cases of diagnostic ambiguity or functional tremor [10,72,148].

The integration of emerging technologies marks a promising frontier in tremor care. AI and machine learning are being applied to neurophysiological and wearable sensor data to enhance diagnostic precision, distinguish overlapping phenotypes, and enable continuous monitoring [150,151,152,153,154,155]. AI-driven algorithms could eventually support real-time symptom tracking and adaptive treatment planning. Wearable systems not only enable remote assessment but also offer therapeutic functions via closed-loop neuromodulation or tremor-suppressing orthotics. Non-invasive stimulation techniques, including transcranial magnetic stimulation and peripheral nerve stimulation, are under active investigation and have shown encouraging results in selected tremor subtypes [167,168,169,170,171,172,173,174].

Nevertheless, the implementation of these technologies faces challenges related to cost, access, and the need for specialised expertise. Ensuring equitable availability of advanced diagnostic and therapeutic modalities remains a critical goal to prevent disparities in tremor management. Future research should also prioritise the identification of robust biomarkers for disease progression and treatment response, especially as precision medicine approaches continue to evolve.

In conclusion, the 2018 consensus criteria mark a critical milestone in the classification and clinical approach to tremor syndromes. This model adopts a syndromic framework and incorporates advanced diagnostic tools, facilitating more individualised and evidence-based care. However, continuous refinement of the classification system and equitable deployment of emerging technologies will be essential to translate these advances into meaningful patient outcomes. AI and other technological innovations are poised to play a transformative role in tremor diagnosis and treatment, ushering in a new era of personalised and precise neurology.

## Figures and Tables

**Table 1:** Diagnostic criteria for essential tremor (2018 consensus).

Feature	Essential Tremor (ET)	ET Plus
Inclusion Criteria	Isolated bilateral upper limb action tremor (postural or kinetic). May also involve tremor in the head, voice, or lower limbs.	Meets all ET criteria, plus presence of additional neurological signs of uncertain significance.
Duration	≥3 years	
Associated Signs	No other neurologic signs (such as dystonia, ataxia, or parkinsonism).	Presence of soft neurologic signs such as the following: Questionable dystonic posturing. Impaired tandem gait. Questionable cognitive impairment. Mild rest tremor.
Exclusion Criteria	Other neurologic signs.	Hard neurologic signs (e.g., clear dystonia, parkinsonism).
	Isolated focal tremors (voice, head). Orthostatic tremor with a frequency > 12 Hz. Task- and position-specific tremors. Sudden onset and step-wise deterioration. Evidence of functional origin. Tremor attributable to another condition or drug.	

**Table 2:** Pharmacological treatments for essential tremor, classified by the level of recommendation (American Academy of Neurology).

Level of Recommendation	Drug	Recommended Dose (mg/Day)	Type
A	Propranolol	120–320	Beta-blocker
	Primidone	37.5–750	Antiepileptic
B	Nadolol	120–240	Beta-blocker
	Atenolol	100	Beta-blocker
	Sotalol	160	Beta-blocker
	Topiramate	50–200	Antiepileptic
C	Zonisamide	100–200	Antiepileptic
	Flunarizine	10–20	Calcium channel blocker
	Nicardipine	40–120	Calcium channel blocker
	Nimodipine	60–240	Calcium channel blocker
	Alprazolam	0.75	Benzodiazepine
	Clonazepam	0.25–5	Benzodiazepine
	Olanzapine	2.5–10	Neuroleptic
	Clozapine	12.5–75	Neuroleptic
U	Timolol	10	Beta-blocker
	Metoprolol	100–200	Beta-blocker
	Indenolol	40–80	Beta-blocker
	Arotinolol	10–30	Beta-blocker
	Phenobarbital	50–200	Antiepileptic
	Gabapentin	300–2400	Antiepileptic
	Levetirazetam	500–2000	Antiepileptic
	Pregabaline	150–600	Antiepileptic
	Lacosamide	100–400	Antiepileptic
	Perampanel	4	Antiepileptic
	Methazolamide	100–200	Carbonic anhydrase inhibitor
	Acetazolamide	250–750	Carbonic anhydrase inhibitor

A: *Should be offered.* Established as effective based on at least two consistent Class I studies. B: *Should be considered.* Probably effective based on at least one Class I study or two consistent Class II studies (randomised controlled trials with some methodological limitations). C: *May be considered*. Possibly effective based on at least one Class II study or two consistent Class III studies (controlled studies with lower methodological quality). U: *Insufficient evidence*.

## Abbreviations

The following abbreviations are used in this manuscript:

ADL	Activities of Daily Living
aDBS	Adaptive Deep Brain Stimulation
AI	Artificial Intelligence
CBD	Corticobasal Degeneration
CD	Cervical Dystonia
CIDP	Chronic Inflammatory Demyelinating Polyneuropathy
DBS	Deep Brain Stimulation
EMG	Electromyography
EPT	Enhanced Physiological Tremor
ePT	Essential Palatal Tremor
ET	Essential Tremor
FES	Functional Electrical Stimulation
FT	Functional Tremor
FXTAS	Fragile X-associated Tremor/Ataxia Syndrome
GABA	Gamma-aminobutyric acid
GPi	Globus Pallidus Internus
GWAS	Genome-Wide Association Study
HCT	Hereditary Chin Tremor
HOD	Hypertrophic Olivary Degeneration
HT	Head Tremor
IMU	Inertial Measurement Unit
IHT	Isolated Head Tremor
M1	Primary Motor Cortex
MeSH	Medical Subject Headings
MRgFUS	Magnetic Resonance-guided Focused Ultrasound
MRI	Magnetic Resonance Imaging
MRIgLITT	MRI-guided Laser Interstitial Thermal Therapy
MSA	Multiple System Atrophy
NT	Neuropathic Tremor
PAPT	Progressive Ataxia with Palatal Tremor
PD	Parkinson’s Disease
PWT	Primary Writing Tremor
QUEST	Quality of Life in Essential Tremor
rTMS	Repetitive Transcranial Magnetic Stimulation
SD	Spasmodic Dysphonia
SPT	Symptomatic Palatal Tremor
STN	Subthalamic Nucleus
TAWD	Tremor Associated with Dystonia
TETRAS	The Essential Tremor Rating Assessment Scale
TIA	Transient Ischemic Attack
TMS	Transcranial Magnetic Stimulation
VT	Vocal Tremor
WHIGET	Washington Heights-Inwood Genetic Study of Essential Tremor
ZI	Zona incerta
